# Parkin-dependent regulation of the MCU complex component MICU1

**DOI:** 10.1038/s41598-018-32551-7

**Published:** 2018-09-21

**Authors:** Alessandra Matteucci, Maria Patron, Denis Vecellio Reane, Stefano Gastaldello, Salvatore Amoroso, Rosario Rizzuto, Marisa Brini, Anna Raffaello, Tito Calì

**Affiliations:** 1 Department of Biomedical Sciences and Public Health, University “Politecnica delle Marche”, Via Tronto 10/A, 60126 Ancona, Italy; 2 Department of Biomedical Sciences, University of Padova, via U. Basi 58/b, 35131 Padova, Italy; 3 Max Planck Institute for Biology of Aging, Cologne, Germany; 4 Department of Physiology and Pharmacology, Karolinska Institutet, Solnavägen 9, Quarter B5, Stockholm, SE-17165 Sweden; 5 Precision Medicine Research Center, Binzhou Medical University, Laishan District, Guanhai Road 346, Yantai, Shandong Province 264003 China; 6 CNR Neuroscience Institute, via U. Basi 58/b, 35131 Padova, Italy; 7 Department of Biology, University of Padova, via U. Bassi 58/b, 35131 Padova, Italy; 8 Padua Neuroscience Center (PNC), University of Padua, 35122 Padova, Italy

## Abstract

The mitochondrial Ca^2+^ uniporter machinery is a multiprotein complex composed by the Ca^2+^ selective pore-forming subunit, the mitochondrial uniporter (MCU), and accessory proteins, including MICU1, MICU2 and EMRE. Their concerted action is required to fine-tune the uptake of Ca^2+^ into the mitochondrial matrix which both sustains cell bioenergetics and regulates the apoptotic response. To adequately fulfil such requirements and avoid impairment in mitochondrial Ca^2+^ handling, the intracellular turnover of all the MCU components must be tightly regulated. Here we show that the MCU complex regulator MICU1, but not MCU and MICU2, is rapidly and selectively degraded by the Ubiquitin Proteasome System (UPS). Moreover, we show that the multifunctional E3 ubiquitin ligase Parkin (*PARK2*), whose mutations cause autosomal recessive early-onset Parkinson’s disease (PD), is a potential candidate involved in this process since its upregulation strongly decreases the basal level of MICU1. Parkin was found to interact with MICU1 and, interestingly, Parkin Ubl-domain, but not its E3-ubquitin ligase activity, is required for the degradation of MICU1, suggesting that in addition to the well documented role in the control of Parkin basal auto-inhibition, the Ubl-domain might exert important regulatory functions by acting as scaffold for the proteasome-mediated degradation of selected substrates under basal conditions, i.e. to guarantee their turnover. We have found that also MICU2 stability was affected upon Parkin overexpression, probably as a consequence of increased MICU1 degradation. Our findings support a model in which the PD-related E3 ubiquitin ligase Parkin directly participates in the selective regulation of the MCU complex regulator MICU1 and, indirectly, also of the MICU2 gatekeeper, thus indicating that Parkin loss of function could contribute to the impairment of the ability of mitochondria to handle Ca^2+^ and consequently to the pathogenesis of PD.

## Introduction

The mitochondrial calcium uniporter (MCU) complex is a Ca^2+^-selective channel of the inner mitochondrial membrane (IMM) composed by pore-forming and regulatory subunits^1^. The pore-forming subunits include the protein necessary and sufficient to mediate Ca^2+^ transport across the IMM, MCU^2–4^, its dominant-negative paralog (MCUb)^5^, and the essential MCU regulator (EMRE)^6^. A regulatory heterodimer composed by the stimulatory component MICU1^7–9^ and the inhibitory component MICU2^10,11^ regulates the opening of the channel shaping both the duration of local and global Ca^2+^ signals to cope with the bioenergetics demand of the cell^12–14^ and with the occurrence of apoptotic processes^9^. In the last few years, most of the efforts were aimed at the identification of the different components of the MCU complex and how they cooperate each other to obtain the tight regulation required to maintain mitochondrial homeostasis. Accordingly, MICU1 stimulates MCU at high [Ca^2+^] thus allowing the rapid Ca^2+^ transport in the matrix when required^7,15^, while MICU2, by inhibiting MCU activity at low [Ca^2+^], counteracts the excessive accumulation of Ca^2+^ in conditions of increased driving force, thus protecting from the processes of Ca^2+^ cycling and matrix overload^10,11^. Two additional components are also part of the complex: MCUb is a dominant-negative pore forming subunit that is included in the multimeric channel and sets the physiological activity of MCU through a novel regulatory mechanism^5^, while EMRE is a regulator of the uniporter channel activity necessary for the interaction of MCU with MICU1 and MICU2^6^ which has also been recently claimed to act as a sensor for matrix [Ca^2+^] to prevent mitochondrial Ca^2+^ depletion^16^. In spite of the extensive knowledge on the gatekeeping mechanisms of the mitochondrial Ca^2+^ channel, how the biogenesis (import and folding) and the turnover (stability and degradation) of the MCU complex components are regulated at the post-translational level is much less clear. A study addressing the biogenesis of MICU1 has shown that the oxidoreductase Mia40 mediates dimerization of MICU1 with MICU2 by introducing intermolecular disulphide bonds and that the resulting heterodimer interacts with MCU in a Ca^2+^-dependent manner^17^.

Parkin (*PARK2*) is a 465-residues multifunctional E3 ubiquitin ligase comprising an N-terminal auto-inhibitory ubiquitin-like domain (Ubl), a unique Parkin-specific domain, two RING domains (RING0, RING1), an in between RING (IBR) domain, and a C-terminal RING domain (RING2) whose mutations are the most common known cause of autosomal recessive early-onset Parkinson’s disease (PD)^18–21^. It is now generally accepted that parkin has a protective role by acting in the regulation of protein quality control, stress-related signalling and proteasome-mediated degradation of selective substrates. In mammalian cells, the cooperation between Parkin and the mitochondrial Ser/Thr protein kinase PINK1 ensures the selective clearance of dysfunctional mitochondria, thus mediating cellular protection through the maintenance of mitochondrial physiology by the process of mitophagy^22^. Accordingly, a number of outer mitochondrial membrane (OMM) proteins, including mitofusins, VDACs and subunits of the translocase of the OMM (TOM) interact with or are ubiquitinated by Parkin during the process of mitochondrial clearance in response to mitochondrial depolarization^23^, suggesting that no specific substrate is required for ubiquitin signalling of mitophagy. However, the finding that loss of PINK1 did not influence basal mitophagy despite disrupting depolarization-induced Parkin activation^24^, suggests that Parkin could participate in maintain mitochondrial integrity by different pathways. Parkin was shown to constitutively associate with the ER and the mitochondrial membranes under basal conditions^25–31^ implying a potential role for this protein in mitophagy-independent functions, such as the modulation of the proteasome activity, of the mitochondrial–ER interactions and calcium crosstalk and the degradation or targeting of specific mitochondrial and/or mitochondria-related proteins. Indeed, cumulating evidence strongly supports a role for Parkin in general protein quality control and ER stress pathways^31–39^ under basal conditions. Thus, the role of Parkin in the cell might not be only restricted to protein turnover under conditions of mitophagy induction but its activation may regulate important mitochondrial as well extramitochondrial functions by participating to physiological protein turnover. Interestingly enough, the Parkin Ubl domain has been shown to interact with the 26 S proteasome’s subunits S5a and Rpn10 to accommodate substrate proteins for degradation and facilitate their ubiquitination in sites that are distant from the Parkin-interacting site^40,41^, suggesting that, when present in multi-domain proteins, Ubl domain might act as docking site to other domains to carry out their tasks on specific target substrates^42^.

The comprehension of post-translational regulation of the MCU complex components, both in terms of turnover and degradation, is essential to fully understand its activity in the control of mitochondrial Ca^2+^ and the related cell functions and it is still poorly investigated. To gain additional insights into these aspects, we assessed the half-life of the MCU complex components MCU, MICU1 and MICU2 upon overexpression in model cells. We have found that MICU1, at variance with MCU, is ubiquitinated by Parkin and rapidly turned-over by the Ubiquitin Proteasome System (UPS). We also provide evidence that Parkin overexpression strongly decreases its basal levels, leaving those of MCU essentially unaffected. Furthermore, the presence of Parkin Ubl-domain, but not of Parkin E3 ubiquitin ligase activity, appears to be an important mediator of this function, suggesting that Parkin Ubl-domain might act as docking intermediate for proteasome-mediated degradation.

Our results indicate that Parkin controls the basal levels of the MCU regulator MICU1 and, since the MICU2 stability strictly depends on that of MICU1^10,11^, Parkin, indirectly also controls MICU2 levels, possibly suggesting that PD-related loss of function mutations in Parkin gene may result in defective mitochondrial assembly of the MCU complex components that in turn could contribute to PD pathogenesis. It is also tempting to speculate that Parkin might act as potential pre-import checkpoint for the correct import and assembly of the MCU complex components by tightly controlling the stoichiometry of the complex.

## Results

### Among MCU complex proteins, MICU1 is short-lived and is degraded in a proteasome-dependent manner

**Figure 1:**
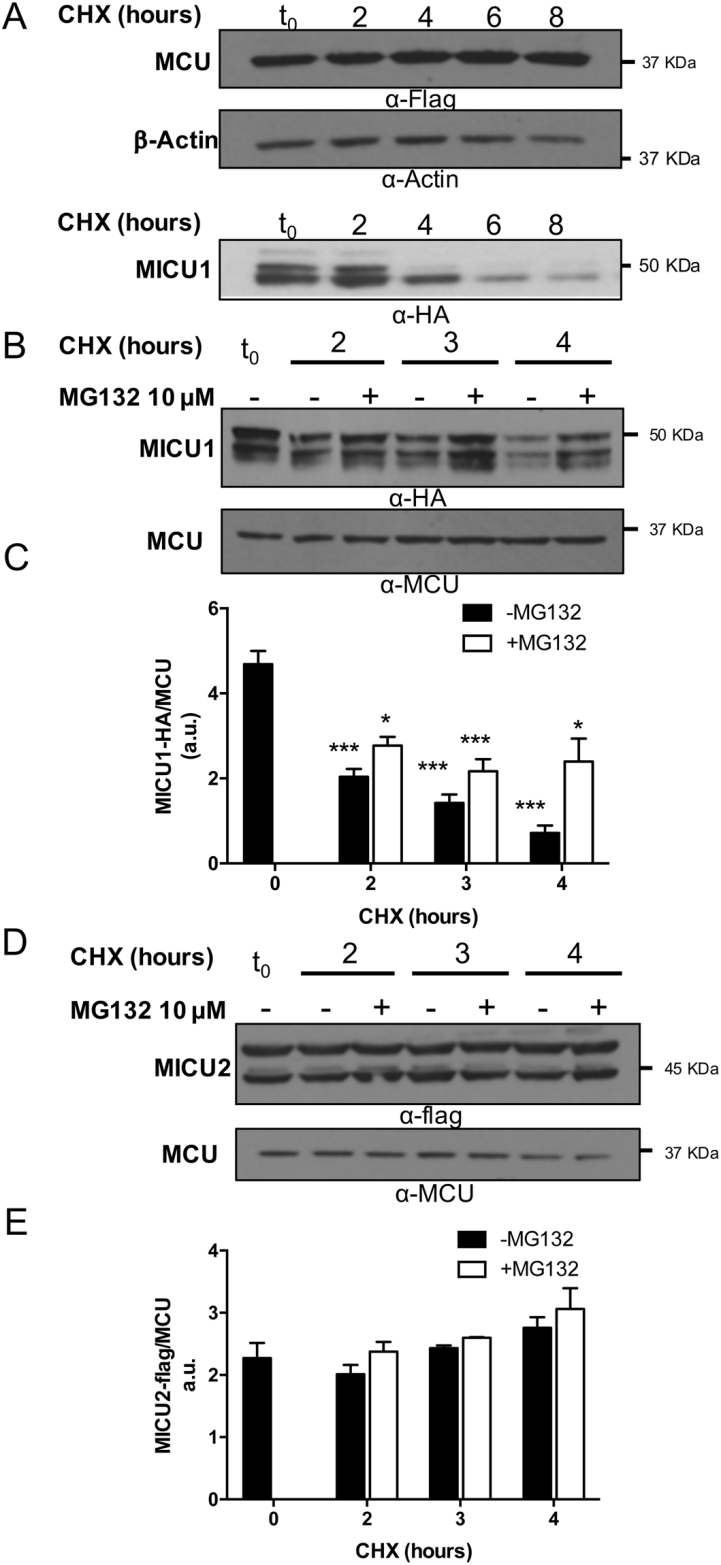
Half-life of MCU complex components. HeLa cells were transfected with MICU1-HA, MICU2-flag or MCU-flag. After 24 hours, cells were treated with 100 μg/ml Cycloheximide and either untreated or treated with 10 μM MG123. Cells were harvested after the indicated time points. Total proteins were extracted and subjected to western blot analysis with α-HA, α-flag or α-MCU antibodies. (**A**) Two independent representative western blots showing the half-life of overexpressed MCU-flag (upper panel) and MICU1-HA (lower panel). Actin is shown for comparison in the same blot for MCU. (**B**) Representative western blot and (**C**) densitometric analysis of MICU1 protein levels upon incubation or not with MG132, normalized to endogenous MCU protein. (**D**) Representative western blot and (**E**) densitometric analysis of MICU2 protein levels, normalized to endogenous MCU protein. Each bar represents mean +/− S.E.M of at least three independent experiments; *p < 0.05; ***p < 0.005. a.u.: arbitrary units.

The turnover of the MCU complex components was studied by a cycloheximide (CHX)-chase assay performed in HeLa cells overexpressing MCU, MICU1 and MICU2 proteins. The western blotting analysis shown in Fig. 1A revealed that overexpressed flag-tagged MCU, the pore forming subunit of the complex, is a very long-lived protein (Fig. 1A, upper panel). For comparison, it was also shown the band relative to β-actin whose levels remain stable at the same time points post incubation with CHX. Figure 1A (bottom panel) also shows (CHX)-chase assay performed in HeLa cells overexpressing MICU1: overexpressed MICU1 undergoes to rapid degradation at the same time course in which overexpressed MCU was stable. To better characterize this finding, the stability of the MCU complex regulators MICU1 and MICU2 was assessed in the presence and in the absence of the proteasome inhibitor MG132. Both of them, when overexpressed in HeLa cells, appeared with two predominant bands of about 50 kDa (MICU1-HA in Fig. 1B and MICU2-Flag in Fig. 1D, respectively), likely corresponding to the un-processed precursor which includes the N-terminal mitochondrial targeting sequence (upper band), and a faster migrating band corresponding to the fully processed mitochondrial form (lower band). Intriguingly, compared to MICU2, whose levels remained unaltered during the 4 hours chase time (see the quantification in Fig. 1E, black bars), the half-life of MICU1 was much faster (see the quantification in Fig. 1C, black bars): its intracellular levels drastically dropped by more than 50% between 2 and 3 hours of treatment. The incubation with the proteasome inhibitor MG132 in CHX-supplemented medium selectively and significantly delayed MICU1 degradation (Fig. 1C, white bars), without affecting the levels of MICU2 (Fig. 1E, white bars). These results indicate that MICU1 is rapidly and selectively turned over in a proteasome-dependent manner. The endogenous MCU was shown for comparison and, in line with what observed for the overexpressed flag-tagged MCU, its levels were unaffected during the time course of CHX incubation (Fig. 1B–D, bottom panels), thus confirming that MCU is a long-lived protein and its amount is not affected by changes in the levels of the MICU1 and MICU2 levels, as previously documented^43^. As additional control, to address whether the overexpressed MCU complex components are functional we have performed aequorin-based measurements of mitochondrial Ca^2+^ uptake upon cell stimulation with an InsP_3_-linked agonist (Supplementary Fig. S1) and found that all the MCU complex components are functional in terms of Ca^2+^ handling since in the presence of overexpressed MCU or MICU1 the mitochondrial Ca^2+^ uptake is increased, instead in the presence of overexpressed MICU2 it is reduced, as previously shown^11^.

### MICU1 is stabilized by MICU2 and the MICU heterodimers are degraded regardless proteasome inhibition

**Figure 2:**
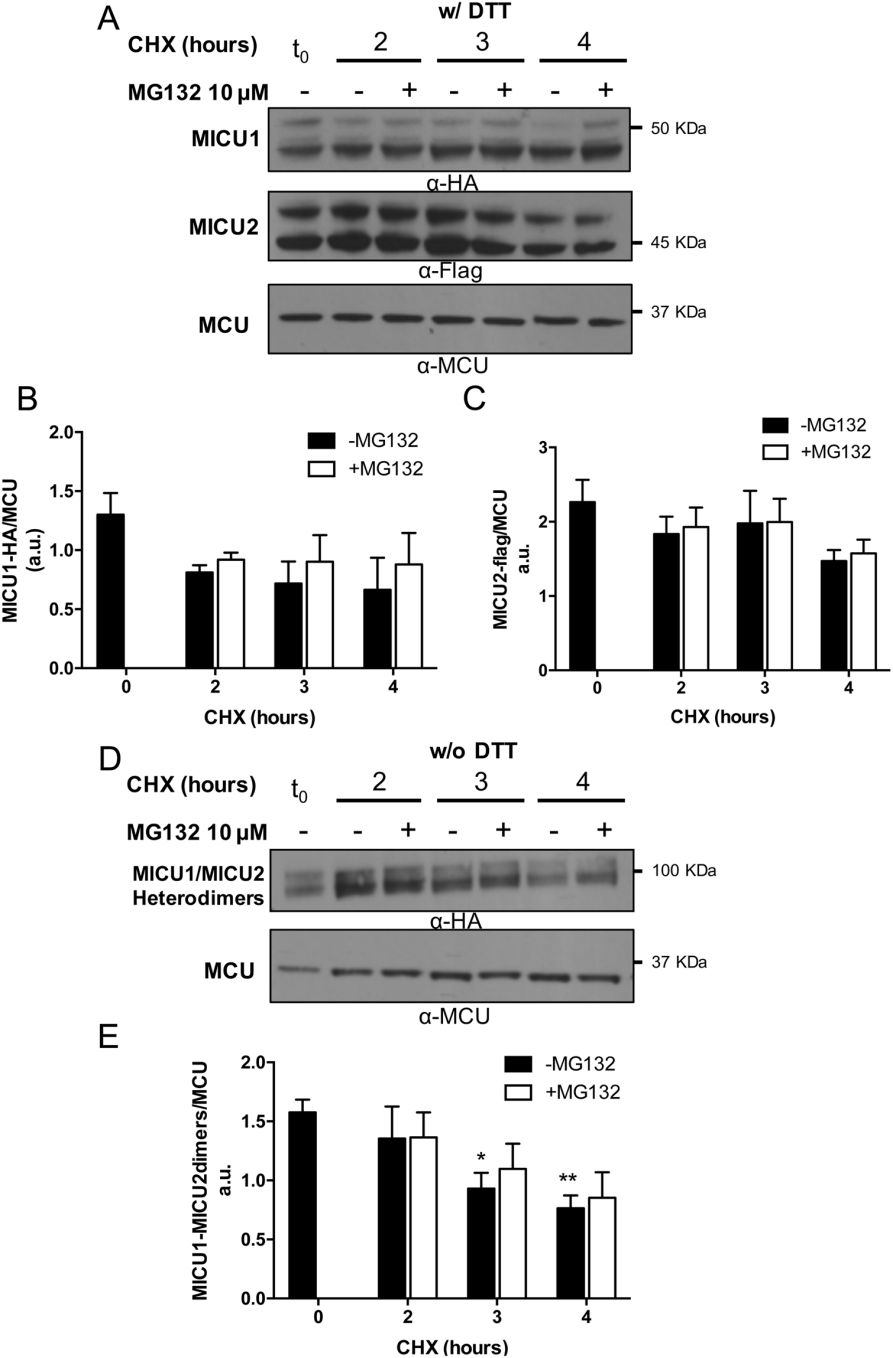
MICU1 is stabilized by MICU2. HeLa cells were transfected with MICU1-HA and MICU2-FLAG. After 24 hours, cells were treated with 100 μg/ml Cycloheximide and either untreated or treated with 10 μM MG123. Cells were harvested and lysed after the indicated time points and subjected to western blot analysis with the indicated antibodies. (**A**) Representative western blot showing the half-life, with or without MG123, upon co-expression of both MICU1 and MICU2. Densitometry analysis of MICU1 (**B**) and MICU2 (**C**) protein levels, normalized to endogenous MCU protein, in cells co-expressing MICU1 and MICU2. (**D**) Non-reducing western blot analysis and (**E**) quantification of the MICU1/MICU2 heterodimers. Each bar represents mean +/− S.E.M of at least three independent experiments; *p < 0.05; **p < 0.01. a.u.: arbitrary units.

In cells, MICU2 forms an obligate heterodimer with MICU1 in the mitochondrial intermembrane space^11^. MICU1 silencing drastically reduces MICU2 levels and abolishes its gatekeeping role, thus causing increases in mitochondrial Ca^2+^ levels both in resting conditions and upon stimulation^7,11,15^. Considering this tight relationship, we decided to assess the stability of MICU1 and MICU2 upon their concomitant overexpression in HeLa cells. We performed gel electrophoresis under reducing and non-reducing conditions to evaluate both monomers and heterodimers half-life (Fig. 2). Interestingly, MICU1 levels remained stable along the chase time (Fig. 2A, top panel) and MG132 treatment did not affected its stability (see quantification in Fig. 2B), supporting the idea that MICU2 increases stability of MICU1, possibly by engaging it in the MICU1/MICU2 complex^44^. According to what shown above, MICU2 half-life remained essentially unchanged (see quantification in Fig. 2C).

Under non-denaturing conditions (Fig. 2D) a doublet at around 90KDa, corresponding to the MICU1/MICU2 heterodimers, is visible and the quantification shown in Fig. 2E indicates that it becomes slightly but significantly reduced at 3 and 4 hours of CHX treatment. However, MG132 incubation was not able to extend the dimer half-life (Fig. 2E). Altogether these data indicate that MICU1 is a short-lived MCU complex regulator that is selectively degraded by the UPS and that co-expression of MICU2 strongly increases its stability.

### MICU1 is Ubiquitinated

**Figure 3:**
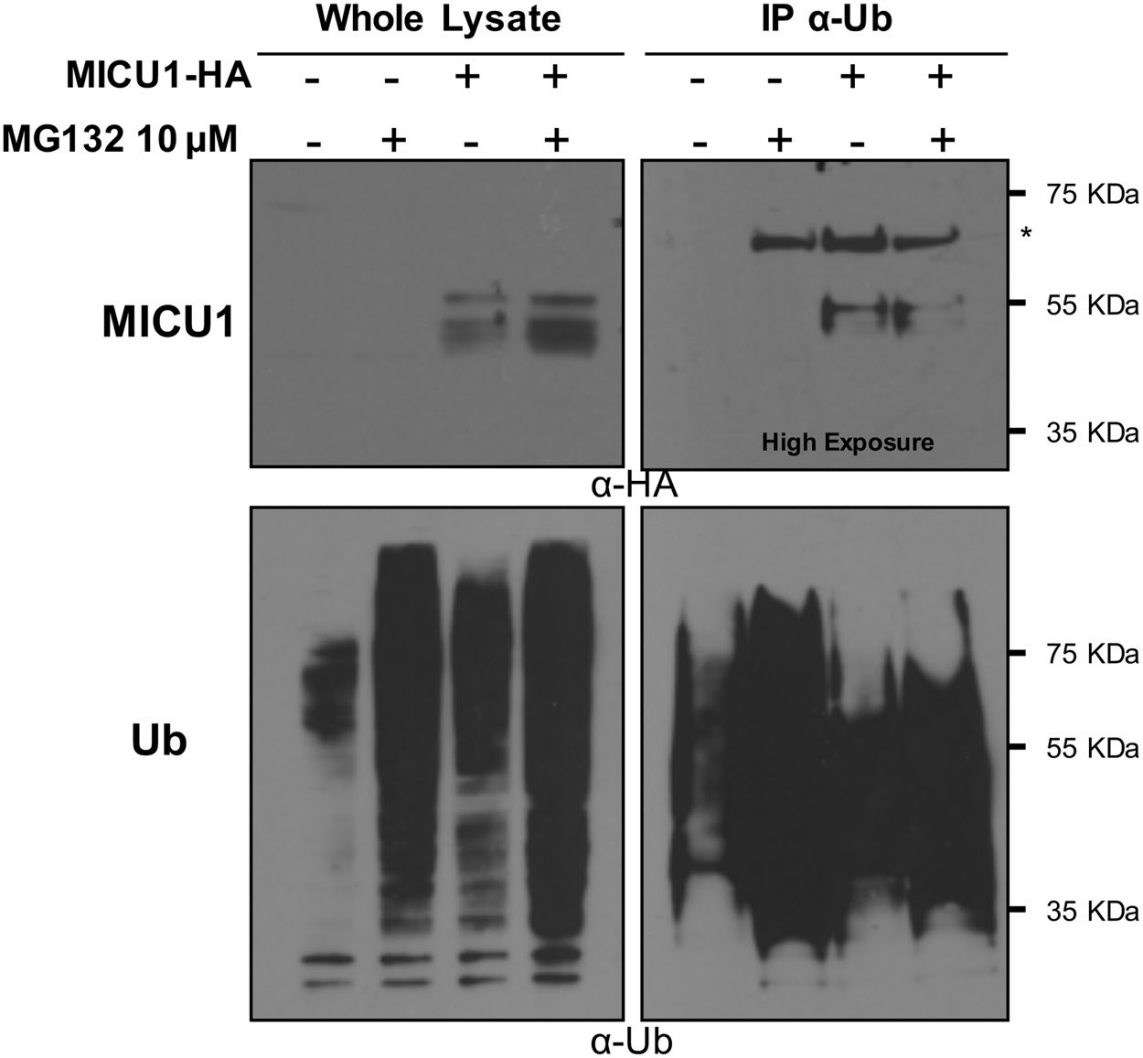
MICU1 is ubiquitinated. HeLa cells were transfected with MICU1-HA or pcDNA3.1 as control. MG132 treatment for 2 hours was used to increase the amount of ubiquitinated proteins. Ubiquitin (Ub) was immunoprecipitated from whole cell lysate with a specific agarose-Tandem Ubiquitin Binding Entities (TUBE). The precipitated proteins were immunoblotted with α-HA to observe the MICU1-HA-Ub complex. Asterisk indicates non-specific bands.

Considering the results described above, we focused on MICU1. To deeply investigate the molecular aspects of its regulation, we checked whether it was subjected to ubiquitination, the first step for UPS degradation. To this aim, we overexpressed HA tagged MICU1 in HeLa cells and either untreated or treated them for 2 hours with 10 μM MG132 before cell lysis. Ubiquitin was then immunoprecipitated from whole cell lysate by specific Agarose-Tandem Ubiquitin Binding Entities (TUBE) and the resulting western blot was probed with an anti-HA antibody to detect the co-immunoprecipitated MICU1. The results of this experiment are shown in Fig. 3. As shown in panel relative to the whole lysate (Fig. 3, top left panel), MG132 treatment strongly increased MICU1 basal level and, as expected, it also increased the total level of ubiquitinated proteins detected by an anti-ubiquitin antibody (Fig. 3, bottom left panel). Immunoprecipitation of the total lysate with TUBE was probed with an anti-HA antibody and we detected a specific 55 KDa migrating band in the MICU1 overexpressing cell lysates corresponding to the MICU1-HA-Ub complex both in the MG132 untreated and treated samples (Fig. 3, top right panel). Remarkably, the immunoprecipitated MICU1 band corresponded to the slow migrating un-processed form, suggesting that a pre-import quality control checkpoint might be in place. The membrane was also probed with an anti-ubiquitin antibody as control and it showed a strong enrichment of the ubiquitinated species as expected (Fig. 3, bottom right panel). These experiments indicate that the pre-import precursor of MICU1 is subjected to tight control by the UPS.

### The Parkinson’s disease related E3 ubiquitin ligase Parkin controls the steady state level of the MCU complex regulators

**Figure 4:**
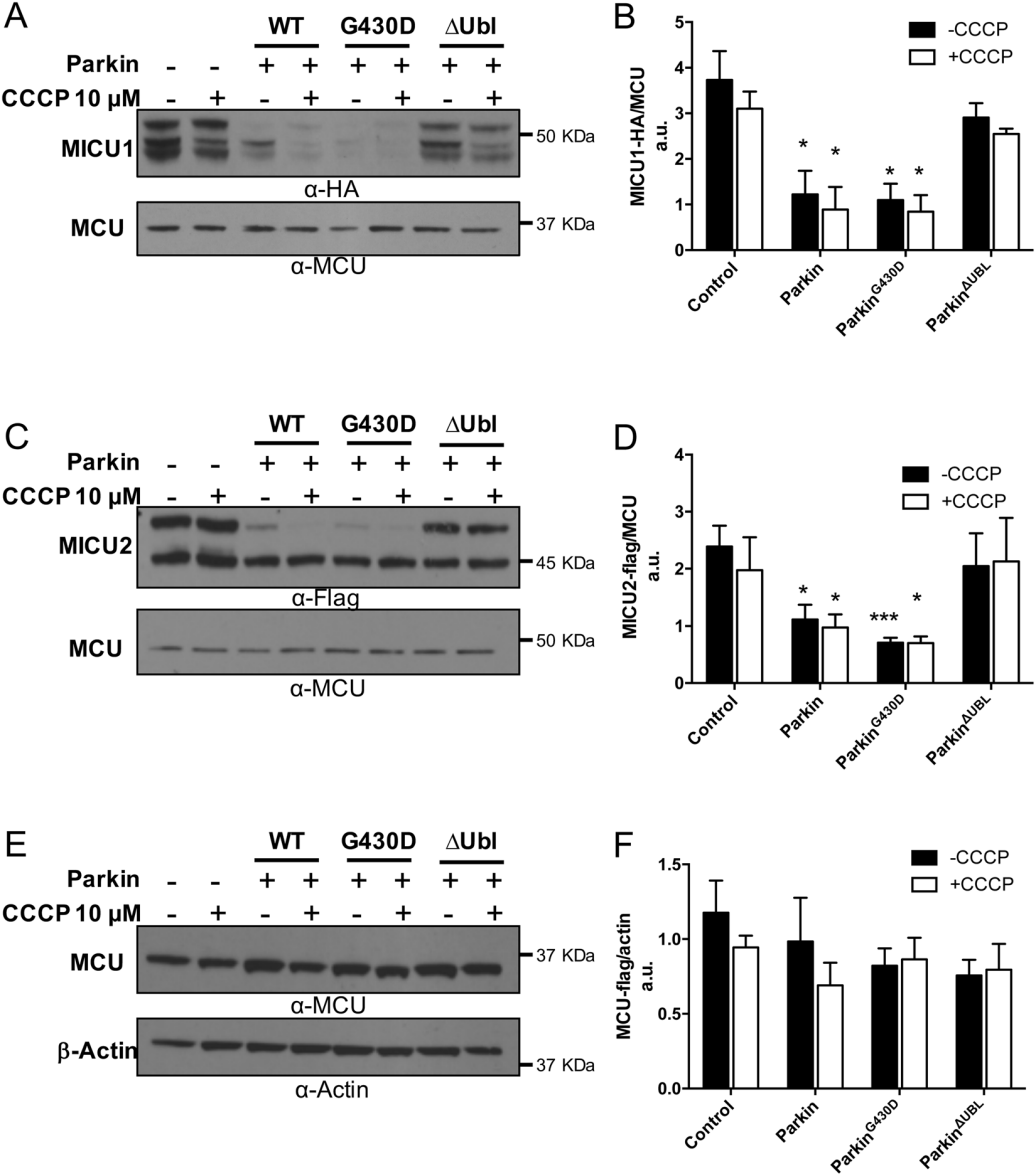
Parkin-dependent regulation of the MCU complex components. HeLa cells were transfected with the indicated Parkin constructs and either MICU1-HA or MICU2-flag. 24 hours after transfectioncells were treated for 2 hours with 10 μM CCCP (where indicated), medium was changed in control groups. Total proteins were extracted and subjected to western blot analysis with the indicated antibodies. Representative western blot image showing the effect of different Parkin constructs (WT, G430D and ΔUbl) on MICU1 (**A**) MICU2 (**C**) and endogenous MCU (**E**) protein levels. Densitometric analysis of MICU1 (**B**) MICU2 (**D**) and endogenous MCU (**F**) protein levels normalized to endogenous MCU protein, in cells co-expressing MICU1-HA or MICU2-flag along with the indicated Parkin constructs. Each bar represents mean +/− S.E.M of at least three independent experiments; * p < 0.05; *** p < 0.005. a.u.: arbitrary units.

In search of a potential E3 ubiquitin ligase involved in the regulation of the basal level of the MCU complex components, we focused our attention on the PD-related protein Parkin, which, besides its role in the execution of mitophagy, has been shown to regulate the level of several proteins involved in the quality control of different mitochondria-related activities, such as biogenesis, integrity, respiration and calcium homeostasis^28–30,45^. To this aim, we transiently expressed flag-tagged MCU, HA-tagged MICU1 and Flag-tagged MICU2 in HeLa cells either alone or together with WT Parkin or the PD-related mutant G430D (characterized by abolished *in vitro* and *in vivo* ligase activity, due to the destabilization of the reactive cysteine C431)^46–50^, or the ΔUbl Parkin mutant (missing the Ubl domain)^51–54^. We thus analysed their expression levels in cells either treated or untreated for 2 hours with CCCP, i.e., a well-established protocol to induce Parkin recruitment to mitochondria and mitophagy activation^55^ and as shown in Supplementary Fig. S2. As shown in Fig. 4A, overexpression of either WT or G430D mutant Parkin induced a marked reduction of the steady-state levels of HA-tagged MICU1 (i.e., of both the slow and fast migrating bands) while no effect is observed when ΔUbl Parkin mutant was overexpressed (see quantification in Fig. 4B performed on the total amount of MICU1). Furthermore, the blot shows that CCCP treatment does not contribute to affect basal levels of MICU1. Altogether, these data suggest that Parkin action on MICU1 level occurs under basal non-depolarizing conditions (and thus presumably independently from its recruitment to mitochondria) but requires the presence of Ubl domain (see quantification in Fig. 4B). Interestingly enough, back-transfection of MICU1 and ∆Ubl-Parkin co-overexpressing HeLa cells with a construct encoding the Ubl domain of Parkin, increased the MICU1 degradation rate (Supplementary Fig. S3). To further confirm the regulatory role of Parkin on the MICU1 turnover rate, we transfected HeLa cells with increasing amounts of Parkin expression vector and performed a Western Blot analysis in order to assess the levels of endogenous MICU1 (Supplementary Fig. S4). Interestingly, a dose-dependent reduction of the endogenous levels of MICU1 (Supplementary Fig. S4 middle left panel) is observed in the presence of overexpressed parkin. The blot also shows that MG132 treatment prevents the decrease (Supplementary Fig. S4, middle right panel).

Figure 4C and D show the analysis of MICU2 levels: the slow migrating non-imported precursor of flag-tagged MICU2 was selectively affected by the overexpression of either WT or the G430D mutant Parkin and, as for MICU1, the ΔUbl Parkin mutant has no effect. Indeed, the MICU2 fast migrating band as well as the overexpressed Flag-tagged MCU (see Fig. 4E and F), are not affected by Parkin overexpression thus highlighting the specificity of the effects observed for MICU1. The observed phenotype could be explained by the fact that the MICU2 stability is dependent on the MICU1 levels and, since MICU2 is a long-lived regulator, as shown in Fig. 1D, the newly synthesized precursor of MICU2 is affected by the Parkin-induced reduction of the MICU1 levels while its mitochondrial mature form remained stable. The quantification shown in Fig. 4D was made taking into account both the bands, and thus shows a reduction of MICU2 levels. None of the conditions tested was significantly affected by CCCP treatment, suggesting that the effect of parkin on MICU1 and MICU2 is independent from mitophagy induction and mitochondrial Parkin recruitment.

### Parkin affects the stability of the co-expressed MICU1 and MICU2 monomers and that of the MICU heterodimers

**Figure 5:**
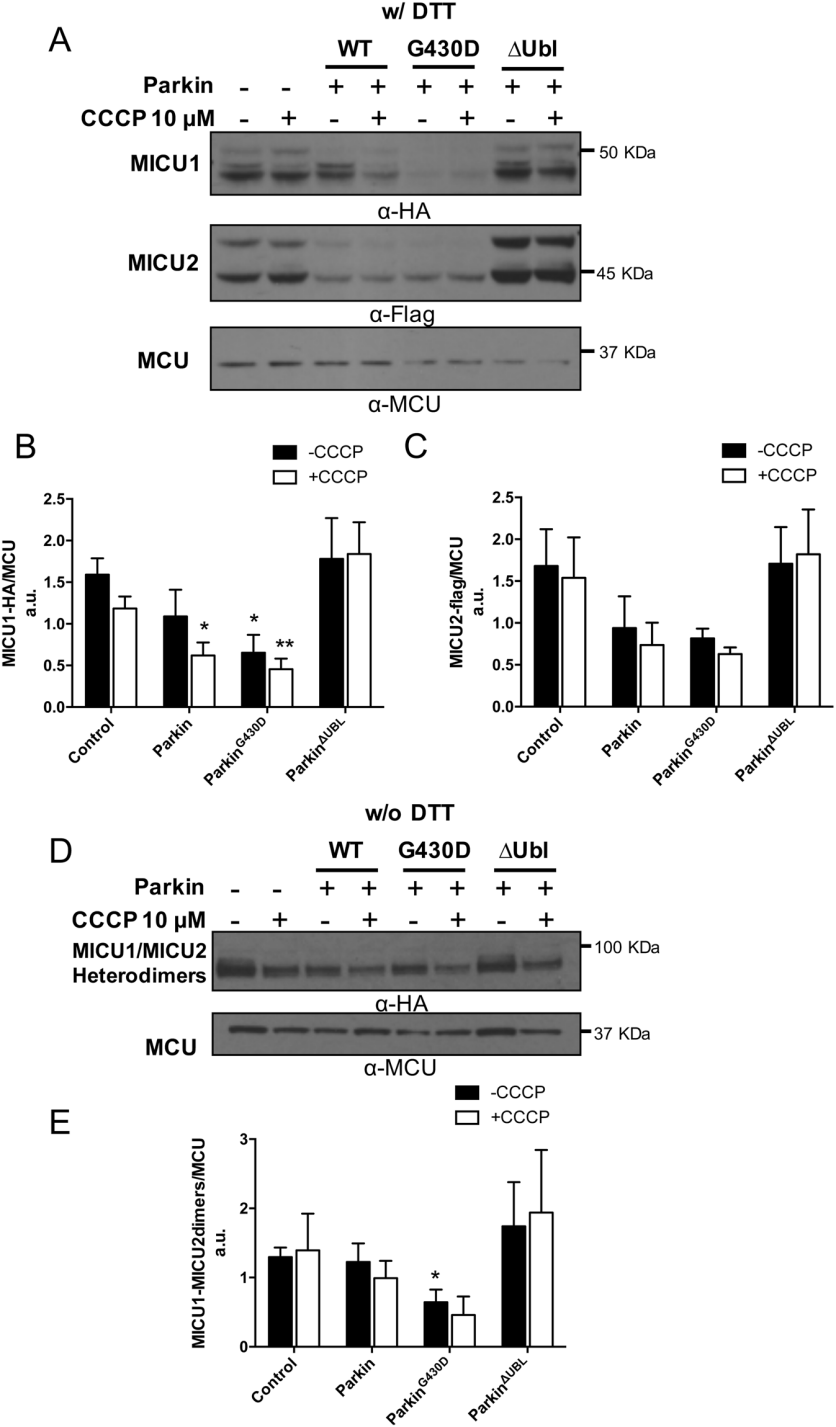
Parkin affect the stability of the MICUs monomers and MICU1/MICU2 heterodimers. HeLa cells were co-transfected with MICU1-HA, MICU2-FLAG and the indicated Parkin constructs. 24 hours later cells were either untreated or treated with 10 μM CCCP for 2 hours (where indicated). Cells were then harvested, lysed and subjected to western blot analysis with the indicated antibodies. (**A**) Representative western blot showing the half-life of co-expressed MICU1 and MICU2, with or without CCCP treatment. Densitometry analysis of MICU1 (**B**) and MICU2 (**C**) protein levels, normalized to endogenous MCU protein, in cells co-expressing MICU1 and MICU2. (**D**) Non-reducing western blot analysis and (**E**) quantification of the MICU1/MICU2 heterodimers in the presence of the indicated Parkin constructs. Each bar represents mean +/− S.E.M of at least three independent experiments; *p < 0.05; **p < 0.01. a.u.: arbitrary units.

As in Fig. 2, we assessed the effect of Parkin on the stability of the co-expressed HA-tagged MICU1 and the Flag-tagged MICU2 monomers as well as that of the MICU heterodimers by performing Western Blot analysis under reducing and non-reducing conditions, respectively (Fig. 5). As already shown in Fig. 2, upon co-expression a shift towards the faster migrating bands of MICU1 and MICU2, corresponding to the mature mitochondrial proteins, is observed when the gel electrophoresis was performed under denaturing conditions, (compare Fig. 4A and C with Fig. 5A, top and middle panels). Interestingly, Parkin overexpression strongly reduced the basal levels of the exogenous co-expressed MICU1 and MICU2, suggesting that it might act upstream before the formation of the more stable MICU1/MICU2 complex (Fig. 2). As shown in Fig. 5A top panel and quantified in 5B, the E3-ubiquitin ligase activity of Parkin was dispensable while the Parkin Ubl domain was required since the mutant lacking this domain was ineffective in decreasing the MICU1 basal levels. Under these conditions, the WT and G430D mutant Parkin, but not the ΔUbl Parkin mutant, also affected the stability of both the mature and immature (pre-import) form of MICU2 (Fig. 5A, middle panel and relative quantification in 5 C). Endogenous MCU levels are unchanged and documented that the decrease observed in MICU1 and MICU2 levels was not due to mitochondria reduction as a consequence of mitophagy activation upon parkin overexpression (Fig. 5A, bottom panel). The level of the MICU1/2 heterodimers (Fig. 5D) was not affected by parkin WT or ΔUbl mutant overexpression, probably due to their increased stability under conditions of co-expression but surprisingly, it is reduced in the presence of the G430D parkin mutant, (Fig. 5E). Treatment with CCCP had no effect on the levels of MCU and MICU1/MICU2 monomers or heterodimers, again suggesting that Parkin can play additional E3-ubiquitin ligase independent functions under basal auto-inhibited state. Due to its great stability and Parkin-insensitivity, endogenous MCU was used as a control.

### MICU1 physically interacts with Parkin

**Figure 6:**
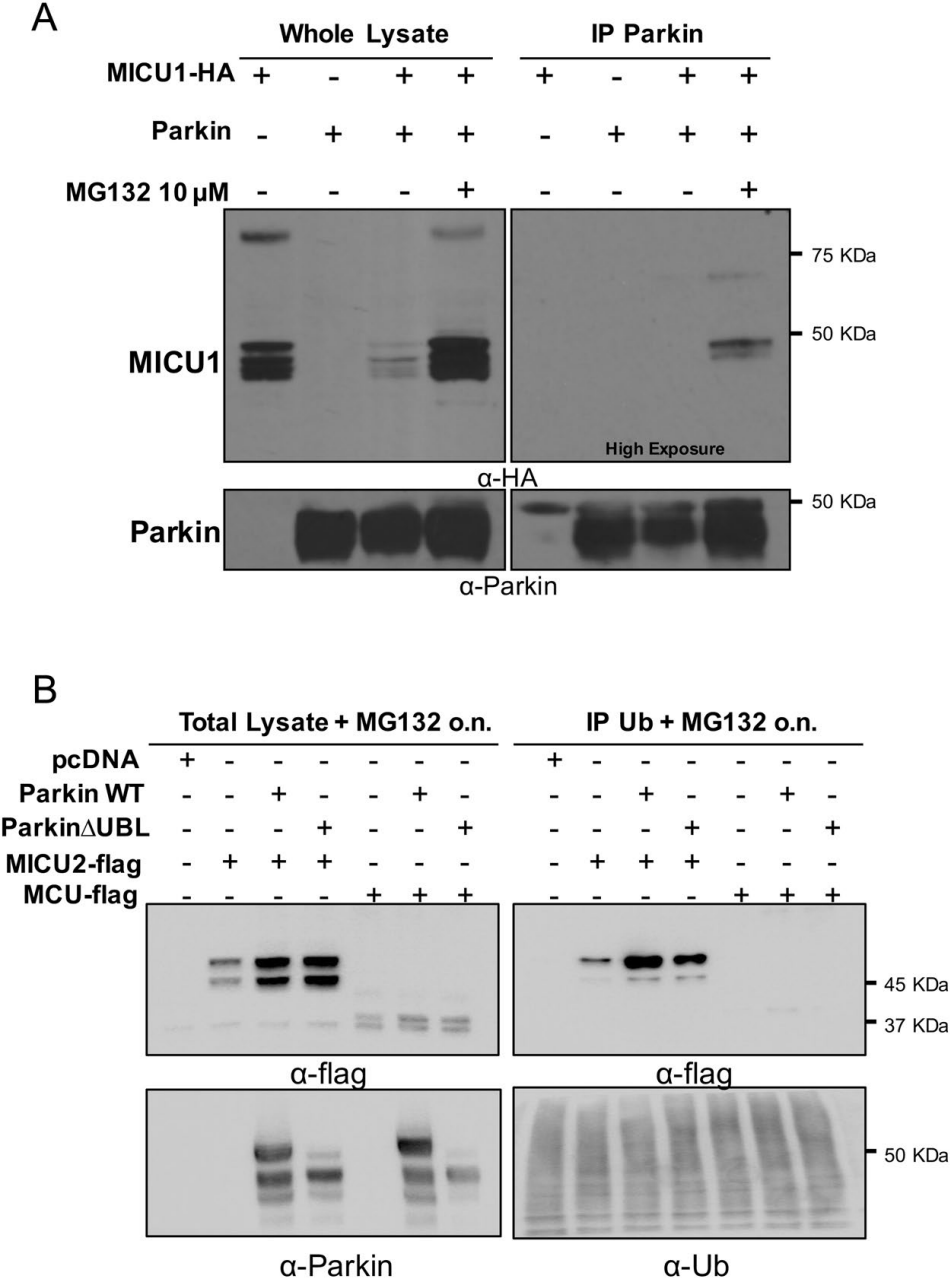
Co-immunoprecipitation analysis of Parkin and the MCU complex components. HeLa cells were transfected with the indicated constructs. Overnight treatment with MG123 was used to prevent Parkin-mediated degradation of MICU1-HA. Parkin was immunoprecipitated from whole cell lysate with anti-Parkin antibody. (**A**) The precipitated proteins were immunoblotted with αHA to observe the MICU1-Parkin complex. (**B**) Ubiquitinated proteins were immunoprecipitated (right panel) from the total lysate (left panel) of HeLa cells overexpressing the indicated constructs, subjected to western blot analysis and probed with the indicated antibodies.

To further investigate the role of Parkin in the regulation of the MICU1 levels, we search for their possible physical interaction by co-immunoprecipitation experiments. Whole cell lysate from HeLa cells expressing MICU1-HA either alone or in combination with Parkin was subjected to immunoprecipitation with an anti-Parkin antibody conjugated agarose matrix. Since Parkin overexpression strongly reduced the basal level of MICU1 (Figs 4A and 6), the cells co-expressing MICU1 and Parkin were also treated with MG132. As shown in Fig. 6A, the western blot of the total lysate decorated with an anti-HA antibody and an anti-Parkin antibody confirmed that both proteins were efficiently expressed and that the reduced level of MICU1 in the presence of WT-Parkin could be efficiently restored by treatment with MG132 (Fig. 6A, left panels). Immunoprecipitated Parkin was then blotted and probed with an anti-HA antibody and a specific band around 50 kDa, corresponding to the slower migrating band of MICU1, was detected only in the co-expressing cells treated with MG132 (Fig. 6A, right panels). This experiment indicates that Parkin physically interacts with the pre-import precursor of MICU1 and, together with the experiments shown above, suggests that Parkin tightly controls the steady-state levels of the MCU regulators before their mitochondrial import by regulating their proteasome-mediated degradation. We also checked whether the ΔUbl Parkin mutant was still able to interact with MICU1 and, as shown in Supplementary Fig. S5, we were still able to pull down MICU1 by immunoprecipitating the Parkin ΔUbl mutant, suggesting that Ubl domain is not required for MICU1-parkin interaction, but, considering the inability of this mutant to increase the MICU1 turnover, it could be essential to proper positioning MICU1 to be degraded as previously suggested for other proteins.

Lastly, since we have observed an effect of Parkin on the MICU2 stability as well, we decided to assess whether it is also subjected to ubiquitination. To this aim, we overexpressed flag tagged MICU2 in the presence or in the absence of either WT or ΔUbl Parkin mutant in HeLa cells and treated overnight with 10 μM MG132, then ubiquitin was immunoprecipitated from whole cell lysate by specific TUBE and the resulting western blot was probed with an anti-flag antibody to detect the co-immunoprecipitated MICU2. As shown in Fig. 6B, MICU2 and Parkin were efficiently expressed and MICU2 levels increased upon MG132 treatment (Fig. 6B, top and bottom left panels, respectively). Immunoprecipitation of the total lysate with TUBE was probed with an anti-flag antibody and showed that the MICU2 precursor was indeed ubiquitinated and, more interestingly, WT Parkin increased its ubiquination (Fig. 6B, top right panel). Of note, MICU2 ubiquitination was only slightly affected upon co-expression with the ΔUbl mutant of Parkin. This finding might be consistent with the fact that Ubl domain of Parkin is dispensable for the observed phenotype. The same experiment was also performed on MCU (Fig. 6B, lanes 5–7) and showed no ubiquitination.

## Discussion

The molecular identification of the components of the mitochondrial Ca^2+^ uniporter has allowed to explore the role of mitochondrial Ca^2+^ and its related activities in diseased states. The MCU complex is in place to prevent channel opening at resting [Ca^2+^], as well as to allow a rapid activation that guarantees mitochondrial Ca^2+^ uptake upon request. Opening of the MCU complex depends on the extra mitochondrial [Ca^2+^] and is mediated by a regulatory dimer formed by the two EF-hand containing proteins MICU1 and MICU2^1^. In the last years, the study of many proteins involved in different forms of neurodegeneration highlighted the importance of mitochondria and of mitochondrial Ca^2+^ uptake in the pathogenesis of the disease^56^. Mitochondrial dysfunction is indeed a common feature of many neurodegenerative diseases, not only of PD but also of Alzheimer’s disease (AD), Amyotrophic Lateral Sclerosis (ALS) and Huntington’s disease (HD). In familial PD, most of the genes identified so far encode proteins that, if mutated, can affect mitochondrial physiology at different levels^57^. Here we show that, the MCU complex regulator MICU1, but not MCU and MICU2, is rapidly and selectively turned-over by the UPS. We also show that the multifunctional E3 ubiquitin ligase Parkin (*PARK2*) is involved in this regulation, i.e., it regulates MICU1 expression levels by a process that is blocked by the proteasome inhibitor MG132. Parkin overexpression strongly reduced basal levels of MICU1 in the absence of CCCP-induced mitochondrial depolarization (Fig. 4A), suggesting that, besides its well-established role in the regulation and activation of mitophagy under conditions of mitochondrial damage, it might participate in protein quality control mechanisms also in the basal state. Under the same conditions, MICU2 stability was also affected (Fig. 4C), probably not through a direct mechanism but because its stability is strongly dependent on the level of MICU1 (but not vice versa)^10,11^. We also found that the precursor of MICU1 is subjected to ubiquitination (Fig. 3), suggesting the existence of a pre-import quality control mechanism involving the UPS. Although recent evidence raised the possibility that selected OXPHOS-related IMS proteins can be ubiquitinated locally^58^, our data are in accordance to the notion that the biogenesis of the mitochondrial intermembrane space proteins is regulated by the cytosolic UPS system^59^. Interestingly, we also found that the action of Parkin is not dependent on its E3-ubiquitin ligase activity since the G430D Parkin mutant still retains the ability to enhance the intracellular turnover of MICU1 (Fig. 4A); rather, the Ubl domain of Parkin seemed to play a major role. Indeed, a ΔUbl Parkin mutant completely lost the ability to regulate the basal levels of MICU1 (Fig. 4A). This finding deserves an explanation: under basal conditions Parkin is in an auto-inhibited state while compelling evidence shows that the ΔUbl mutant exhibits enhanced basal E3 ligase activity compared with the full-length non-phosphorylated Parkin^60–62^. Nonetheless, this mutant completely lost the ability to regulate the basal levels of MICU1, while the WT and the G430D Parkin mutant, which contain this domain, strongly increased the MICU1 intracellular turnover (Fig. 4A). Intriguingly, the reintroduction of the Ubl domain in cells co-expressing the ΔUbl Parkin mutant along with MICU1 was sufficient to restore the ability to increase the degradation rate of MICU1 (Supplementary Fig. S3), suggesting that full length Parkin is required regardless its E3-ubiquitin ligase activity. The precise mechanisms by which this occurs remains unclear, but, accordingly, MICU1 has been found to interact with both WT and the ΔUbl Parkin mutant (Fig. 6 and Supplementary Fig. S5) indicating that the observed effect is “regulatory” and not due to defective interaction possibly dependent on the lack of the Ubl domain. Additionally, Parkin activation by CCCP treatment did not substantially change the effect (Fig. 5A), suggesting that this novel role proposed for Parkin is independent from its recruitment to the outer mitochondrial membrane, but it is rather related to its action at basal auto-inhibited state. We can thus speculate that parkin binding is required for proper MICU1 positioning in order to be ubiquitinated by other ubiquitin ligases and we suggest that Parkin action is part of a post-translation mechanism that controls the amount of MICU1 levels before its import in the intermembrane space and the assembly in the MCU complex. The finding that the Ubl domain of Parkin specifically interacts with the 26 S proteasome for positioning substrate proteins for degradation^40,41^, thus acting as a docking site within multi-domain proteins^42^ is in line with our hypothesis. Furthermore, PINK1 and Parkin have also been shown to regulate the targeting and translation of select nuclear-encoded respiratory chain complexes mRNAs at the mitochondrial outer membrane^63^. Our data are consistent with a role for Parkin and the ubiquitin-proteasome system in selectively regulating the MCU complex regulator MICU1 and, indirectly also MICU2. Interestingly, evidence for an interplay between altered mitochondrial function, impaired mitochondrial Ca^2+^ homeostasis and the MCU complex has also been shown to occur in an *in vivo* model where inhibition of the mitochondrial calcium uniporter was able to rescue dopaminergic neurons in pink1^−/−^ zebrafish^64^ thus, our results enforce the link between altered mitochondrial Ca^2+^ handling and the pathogenesis of PD. Indeed, *PARK2* is the second most common gene mutated in early-onset familial PD^65^, and its mutations have been tightly associated with mitochondrial dysfunction. Parkin dysfunction is also a risk factor for the sporadic form of PD^66^. Loss of Parkin protective function may, together with an impaired mitophagy process, contribute to the dysfunction in autosomal recessive PD. Moreover, emerging evidence also implicates a role for Parkin in AD, ALS and HD^67–69^. The finding that the maintenance of appropriate MICU levels in the mitochondria may be a Parkin-regulated quality control mechanism is thus fundamental not only for the role in the pathogenesis of PD but it might link mitochondrial Ca^2+^ mishandling with the general processes of neurodegeneration. Further studies are required to better understand the precise mechanisms by which Parkin controls the levels of MICU1.

## Methods

### Cell culture, transfection and treatments

HeLa cells were grown in Dulbecco’s modified Eagle’s medium (DMEM) (Invitrogen), supplemented with 10% fetal bovine serum (FBS) (Invitrogen) and transfected with a standard calcium-phosphate procedure as previously described^70^. The following constructs were used: human MICU1, MICU2 and MCU genes tagged at C-term with HA and FLAG sequences; human wt-G430D-∆Ubl-Parkin gene; and Ubl domain tagged with Myc sequence or untagged. All constructs used in this study were cloned into the pcDNA3.1 vector. Mock vector was used as control in all overexpression experiments. In half-life experiments, after transfection of the plasmids for the expression of the tagged proteins, cells were grown in culture media supplemented with Cycloheximide (100 μg/ml), which blocks mRNA translation, for different time periods as indicated. Cells harvested at the starting point have been considered for the basal level of the protein (t0). Stock solutions of Cycloheximide (Sigma-Aldrich) 100 mg/ml in ethanol, MG132 (Calbiochem) 10 mM in DMSO and CCCP (Sigma-Aldrich) 10 mM in ethanol were stored at −20 °C and used at 1000X dilution.

### Western blotting

Whole cell lysates were prepared from HeLa cell resuspended in RIPA buffer (150 mM NaCl, 1% Triton, 1% Na-Deoxycolate, 0.1% SDS, 50 mM Tris-HCl pH 8.0), supplemented with proteases and phosphatases inhibitors (ROCHE) on ice for 30 min. Proteins were cleared by centrifugation at 20000xg for 10 min at 4 °C. Protein concentration was determined by BCA method. Protein extracts were denatured in 1X Laemmli buffer supplemented with 10 mM dithiothreitol (DTT) at 95 °C for 5 min. For non-denaturating condition DTT was not added and samples were boiled at 75 °C for 5 min. Samples were loaded on SDS-polyacrylamide gels and separated by electrophoresis. Proteins were transferred to 0.2 μm nitrocellulose membranes. Membranes were blocked in 5% fat free milk for 1 hour at room temperature, incubated with primary antibody (α-HA, α-FLAG, α-MCU, α-Parkin) overnight at 4 °C and with secondary antibodies for 1 hour at room temperature. Signal was developed by chemiluminescence, bands were visualized by ECL and quantified by ImageJ software. All of the results are expressed as means ± SEM, and Student’s t test was used for the statistics. α-HA, α-FLAG were from Cell Signaling Technology, α-MCU was from Sigma-Aldrich and α-Parkin from Santa Cruz.

### Immunoprecipitation

Whole cell lysates were prepared from HeLa cell resuspended in a Lysis Buffer containing 50 mM HEPES, 150 mM NaCl, 5 mM EDTA, 0.5% DDM, supplemented with proteases and phosphatases inhibitors. Lysates were cleared by spinning at 20000 g for 10 min at 4 °C. Ubiquitinated proteins were immunoprecipitated with agarose-Tandem Ubiquitin Binding Entities (agarose-TUBEs, TebuBio). 500 μg of proteins were incubated with agarose-TUBEs in PBS overnight at 4 °C. Immunoprecipitates were washed with PBS three times and boiled in 30 μl of Laemmli buffer. Parkin was immunoprecipitated using an anti-Parkin antibody (PRK8 Santa Cruz). 500 μg of proteins were incubated overnight with 4 μg of antibody, next day the complexes were incubated 3 hours with Protein G Agarose matrix (GE healthcare) for purification. Immunoprecipitates were washed with 50% lysis buffer in PBS three times and boiled in 30 μl of Laemmli buffer. Total lysates and immunoprecipitates were subjected to western blot assay for detection of the indicated proteins.

### Statistical Analysis

Data was subjected to Student’s t test was used for the statistics. Error bars on all graphs indicate means ± SEM, from replicate experiments. In each instance *p < 0.05; **p < 0.01; ***p < 0.005; no * represents no significance. All statistical analyses and graph production was carried out using GraphPad Prism (version 6, GraphPad Software, La Jolla California USA).

## Electronic supplementary material

Supplementary information + Full length Blots

## Data Availability

All data generated or analysed during this study are included in this published article (and its Supplementary Information files).
