# Publisher Correction: Parkin-dependent regulation of the MCU complex component MICU1

**DOI:** 10.1038/s41598-018-37929-1

**Published:** 2019-03-12

**Authors:** Alessandra Matteucci, Maria Patron, Denis Vecellio Reane, Stefano Gastaldello, Salvatore Amoroso, Rosario Rizzuto, Marisa Brini, Anna Raffaello, Tito Calì

**Affiliations:** 1 Department of Biomedical Sciences and Public Health, University “Politecnica delle Marche”, Via Tronto 10/A, 60126 Ancona, Italy; 2 Department of Biomedical Sciences, University of Padova, via U. Basi 58/b, 35131 Padova, Italy; 3 Max Planck Institute for Biology of Aging, Cologne, Germany; 4 Department of Physiology and Pharmacology, Karolinska Institutet, Solnavägen 9, Quarter B5, Stockholm, SE-17165 Sweden; 5 Precision Medicine Research Center, Binzhou Medical University, Laishan District, Guanhai Road 346, Yantai, Shandong Province 264003 China; 6 CNR Neuroscience Institute, via U. Basi 58/b, 35131 Padova, Italy; 7 Department of Biology, University of Padova, via U. Bassi 58/b, 35131 Padova, Italy; 8 Padua Neuroscience Center (PNC), University of Padua, 35122 Padova, Italy

Correction to: *Scientific Reports* 10.1038/s41598-018-32551-7, published online 21 September 2018

In the original version of this Article, the author Denis Vecellio Reane was incorrectly indexed. This error has now been corrected.

