# Supplementary material for: Parkin-dependent regulation of the MCU complex component MICU1
Source: Sci Rep. 2018 Sep 21;8:14199. doi: 10.1038/s41598-018-32551-7 (PMC6155109; doi:10.1038/s41598-018-32551-7)

Supplementary Information

Parkin-dependent regulation of the MCU complex component MICU1

Alessandra Matteucci[1], Maria Patron[2,3], Denis Vecellio Reane[2], Stefano Gastaldello[4,5], Salvatore Amoroso[1], Rosario Rizzuto[2,6], Marisa Brini[7], Anna Raffaello[2*] and Tito Calì[2,8*]

[1]Department of Biomedical Sciences and Public Health, University "Politecnica delle Marche", Via Tronto 10/A, 60126, Ancona, Italy.
[2]Department of Biomedical Sciences, University of Padova, via U. Basi 58/b, 35141, Padova, Italy.
[3]Max Planck Institute for Biology of Aging, Cologne, Germany.
[4]Department of Physiology and Pharmacology, Karolinska Institutet, Solnavägen 9, Quarter B5, Stockholm, SE-17165, Sweden.
[5]Precision Medicine Research Center, Binzhou Medical University, Laishan District, Guanhai Road 346, Yantai, Shandong Province, 264003 China.
[6]CNR Neuroscience Institute, via U. Basi 58/b, 35141, Padova, Italy.
[7]Department of Biology, University of Padova, via U. Basi 58/b, 35141, Padova, Italy.
[8]Padua Neuroscience Center (PNC), University of Padua, 35122, Padova, Italy.

* Address correspondence to: anna.raffaello@unipd.it or tito.cali@unipd.it

Contents

-Supplementary figures S1 to S5
-Full-Length Blots

Matteucci et al., Figure S1

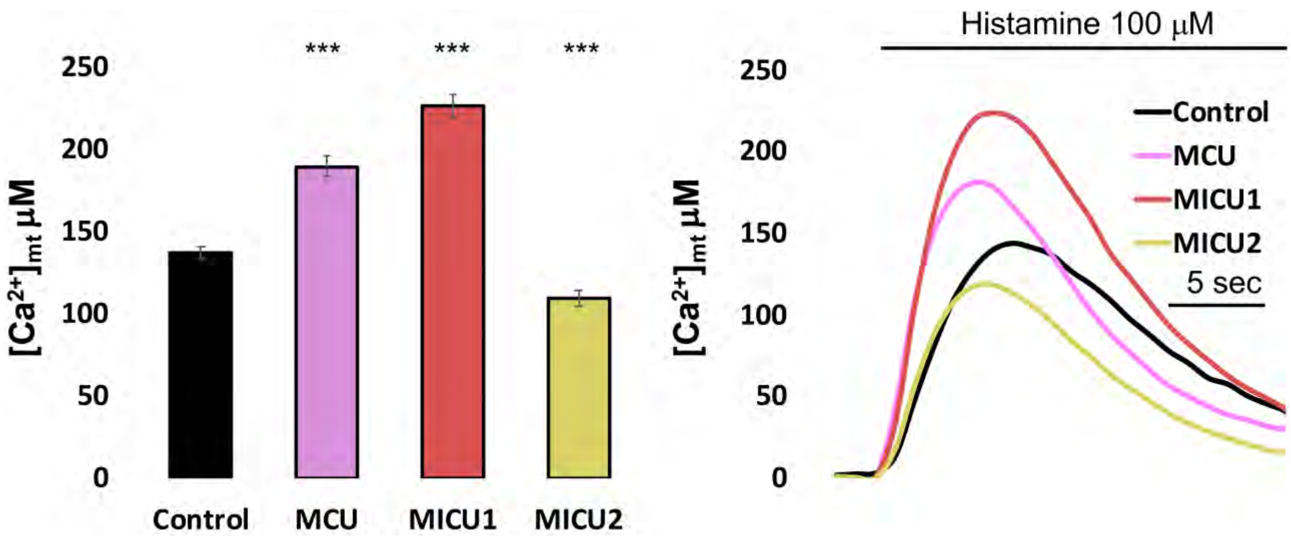

Matteucci et al., Figure S2

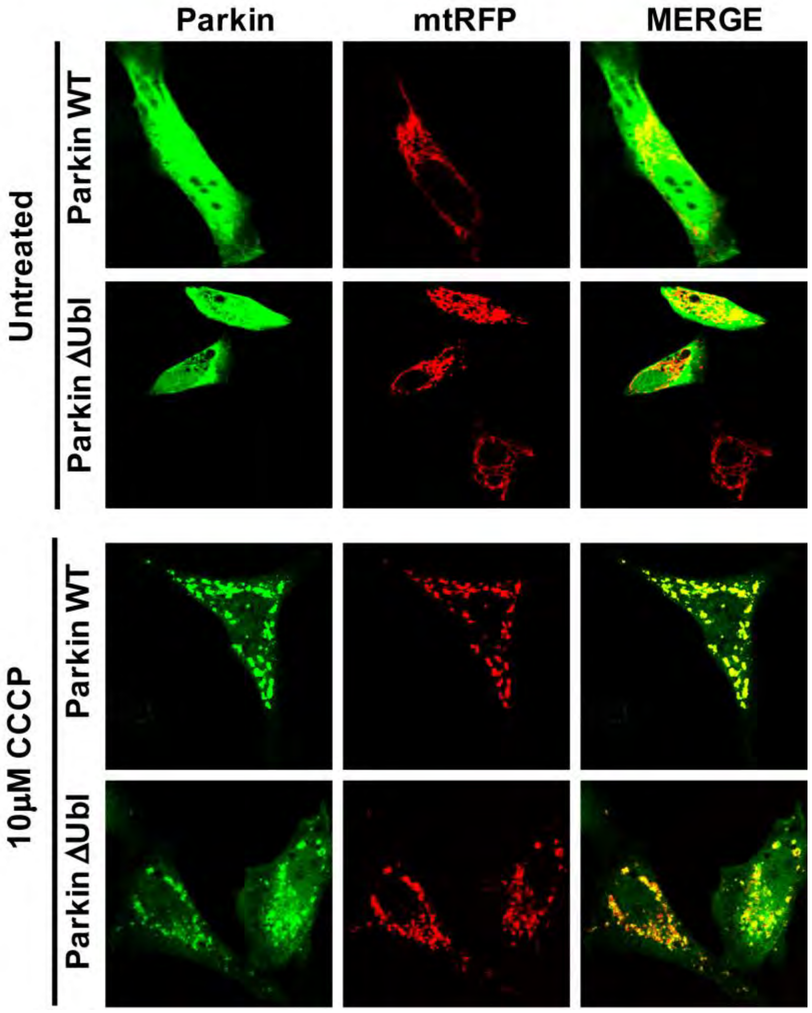

Matteucci et al., Figure S3

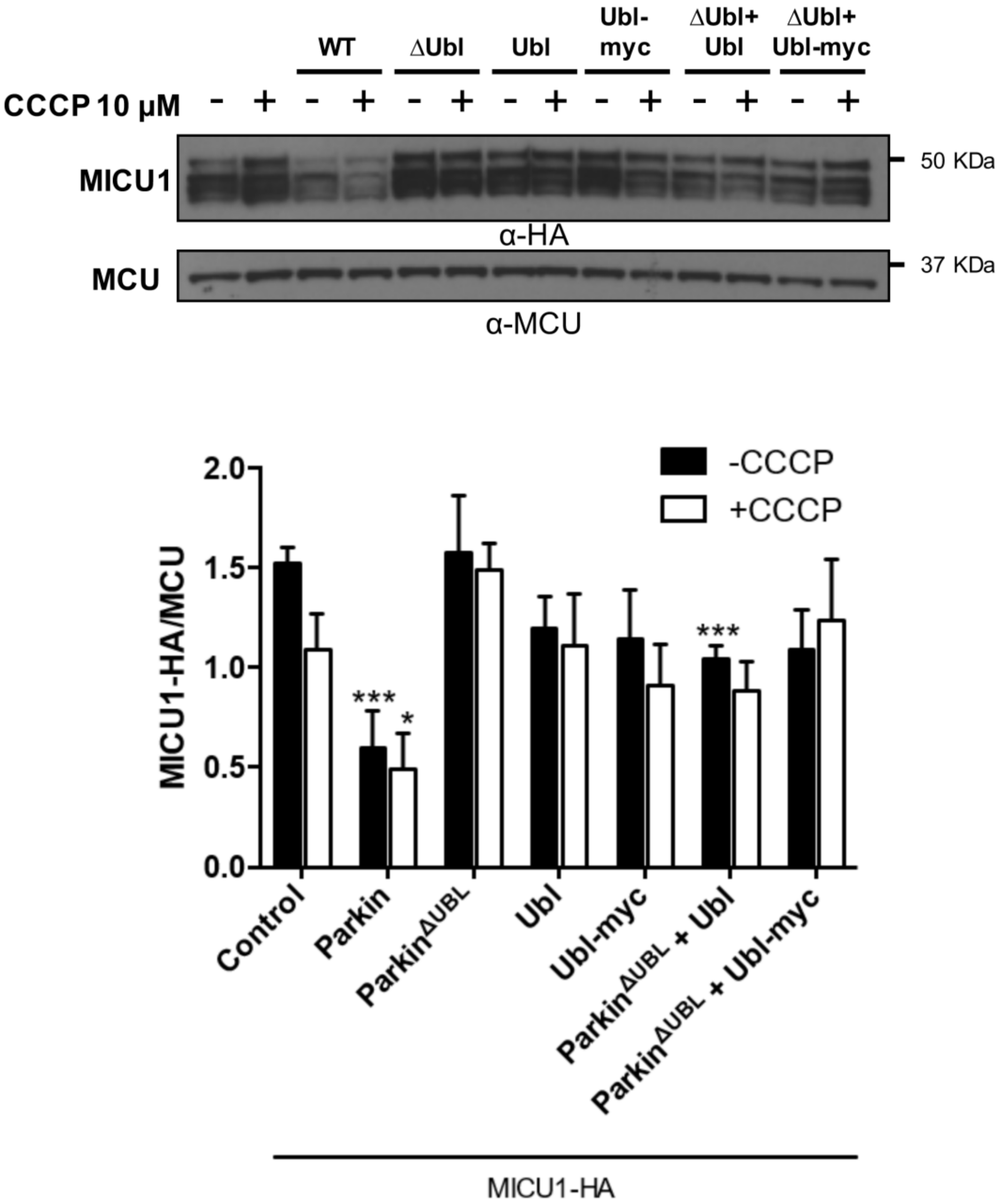

Matteucci et al., Figure S4

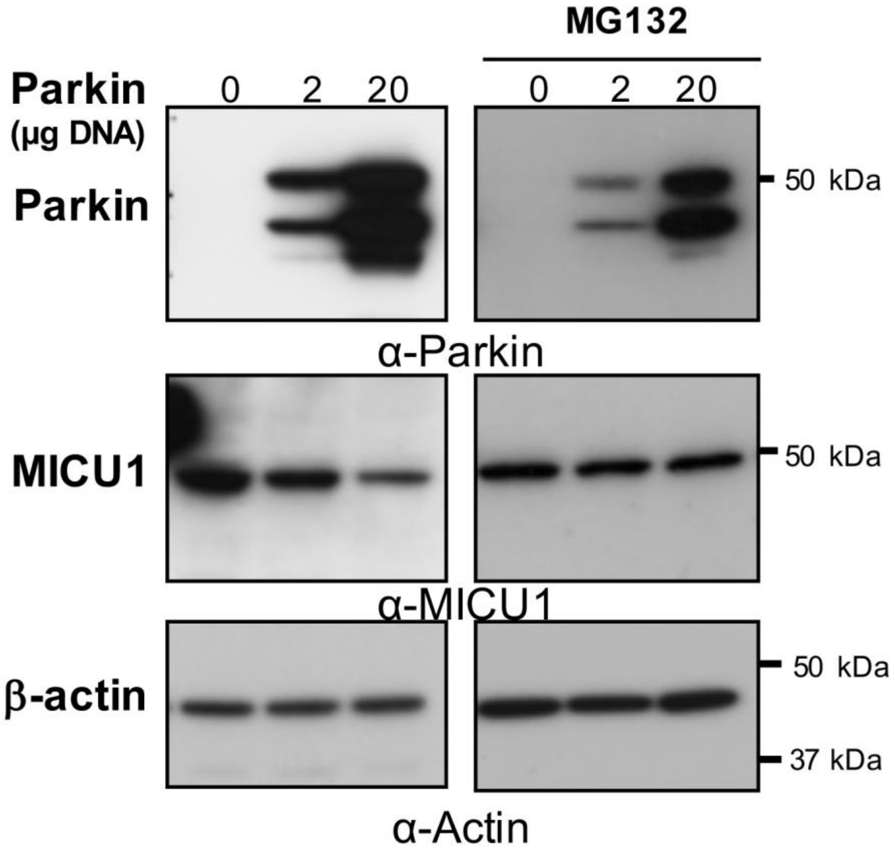

Matteucci et al., Figure S5

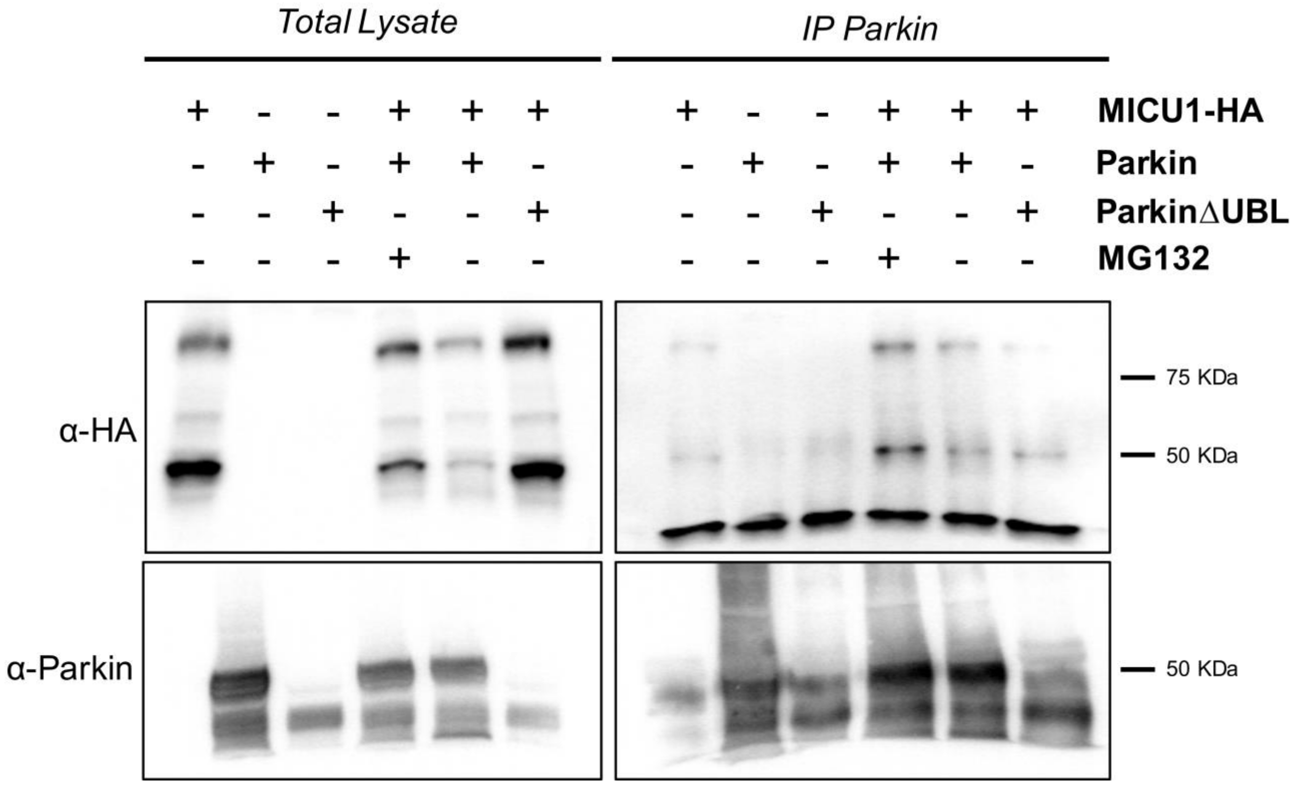

Supplementary Figure Legends

Supplementary Fig. S1 MCU complex components are functional

HeLa cells were transfected with mitochondrial-aequorin either alone or along with the indicated constructs. Mitochondrial Ca^{2+} transients (right panel) are recorded upon cell stimulation with 100 µM histamine. Indicated on the left are the peaks (µM Ca^{2+}) obtained under basal conditions and in the presence of overexpressed MCU, MICU1 and MICU2. *** $p<0.005$.

Supplementary Fig. S2 Mitophagy activation and mitochondrial translocation of Parkin upon CCCP treatment.

HeLa cells were co-transfected with WT or ΔUbl Parkin along with an mtRFP and either left untreated or treated with 10µM CCCP for 2hours to induce Parkin translocation at mitochondria (see merge panels). Parkin was revealed by immunocytochemistry with α-Parkin primary antibody and Alexa Fluor 488 conjugated secondary antibody.

Supplementary Fig. S3 Rescue of defective ΔUbl parkin by expression of Ubl domain

(A) Representative Western blot of HeLa cells co-transfected with MICU1-HA and the indicated constructs. After 24 hours, the cells were treated for 2 hours with 10 µM CCCP (where indicated). Total proteins were extracted and subjected to western blot analysis with the α-HA antibody. (**B)** Densitometric analysis of protein levels, normalized to endogenous MCU protein. Each bar represents mean +/- S.E.M of at least three independent experiments; * $p<0.05$, *** $p<0.005$. a.u.: arbitrary units versus pcDNA

Supplementary Fig. S4 Dose-dependent effect of Parkin on endogenous MICU1

HeLa cell were transfected with 2 or 20 µg (as indicated)) of Parkin expression vector or pcDNA3.1 as control. After 24 hours cells were harvested, total proteins were extracted and subjected to western blot analysis with α-MICU1 and α-MCU antibodies. A representative western blot shows

the dose-dependent effect of WT Parkin overexpression on endogenous MICU1 levels in the presence or absence of MG132.

Supplementary Fig. S5 MICU1 co-immunoprecipitates with Parkin ΔUbl

HeLa cells were transfected with the indicated constructs. Overnight treatment with MG123 was used to prevent the degradation of MICU1-HA upon Parkin overexpression. Parkin was immunoprecipitated from whole cell lysate with anti-Parkin antibody (right panel). The immunoprecipitated proteins from the total lysate (left panel) of HeLa cells overexpressing the indicated constructs were subjected to western blot analysis.

Full-Length Blots

Fig 1 A

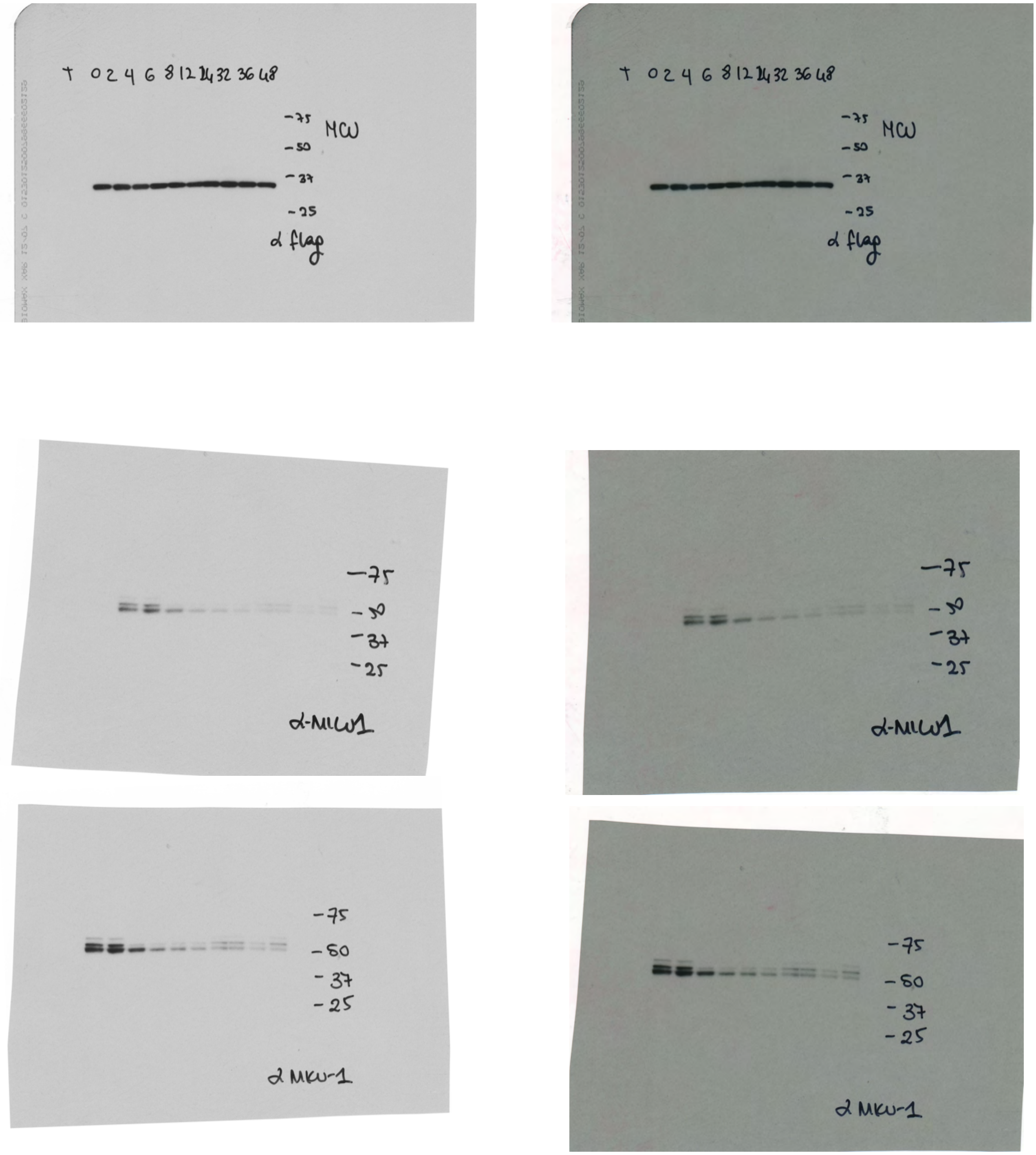

Fig 1 B

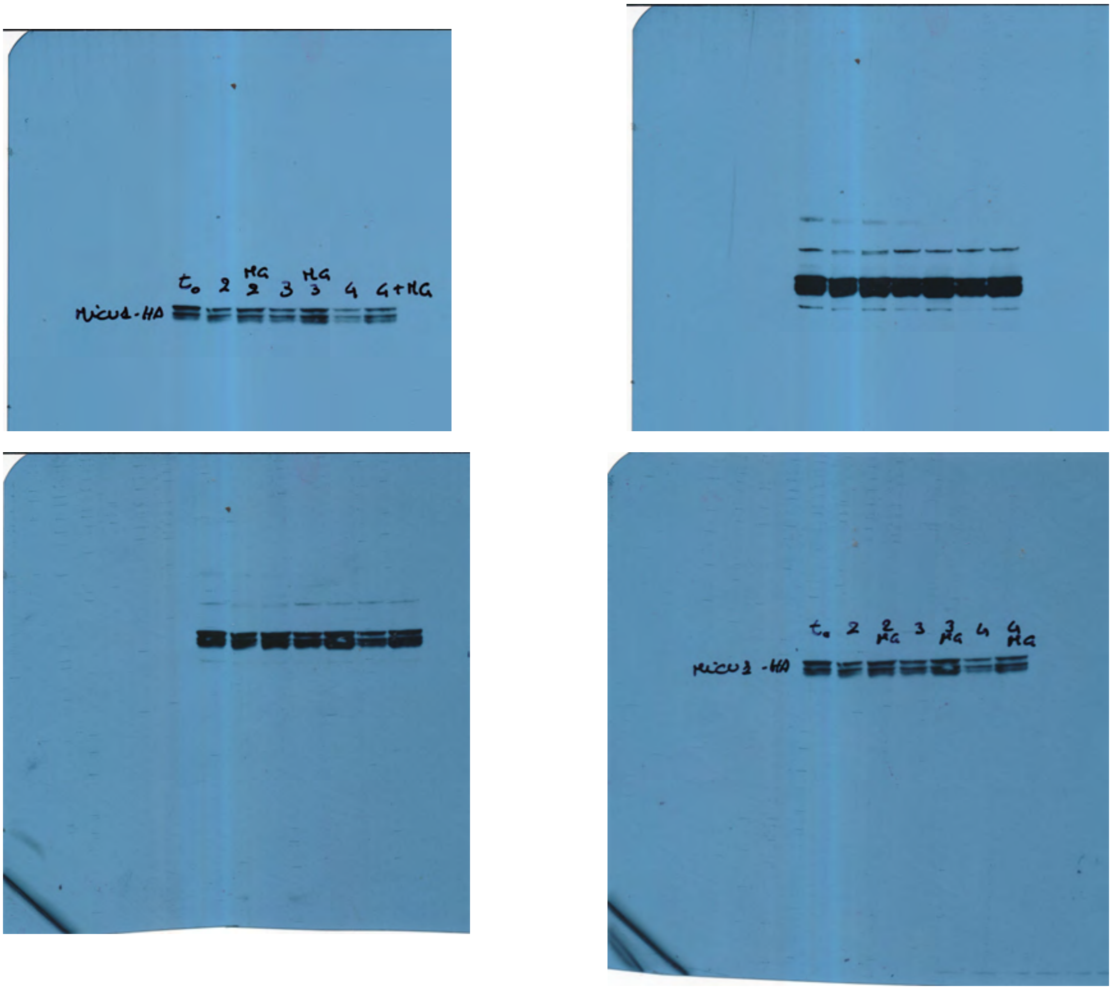

Fig.1B MCU

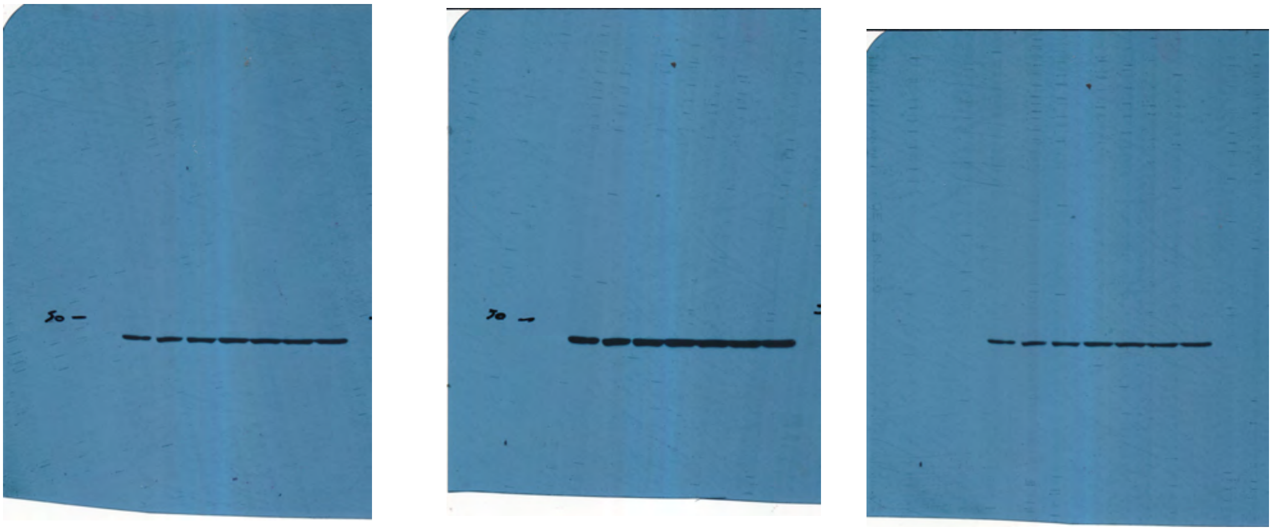

Fig.1D

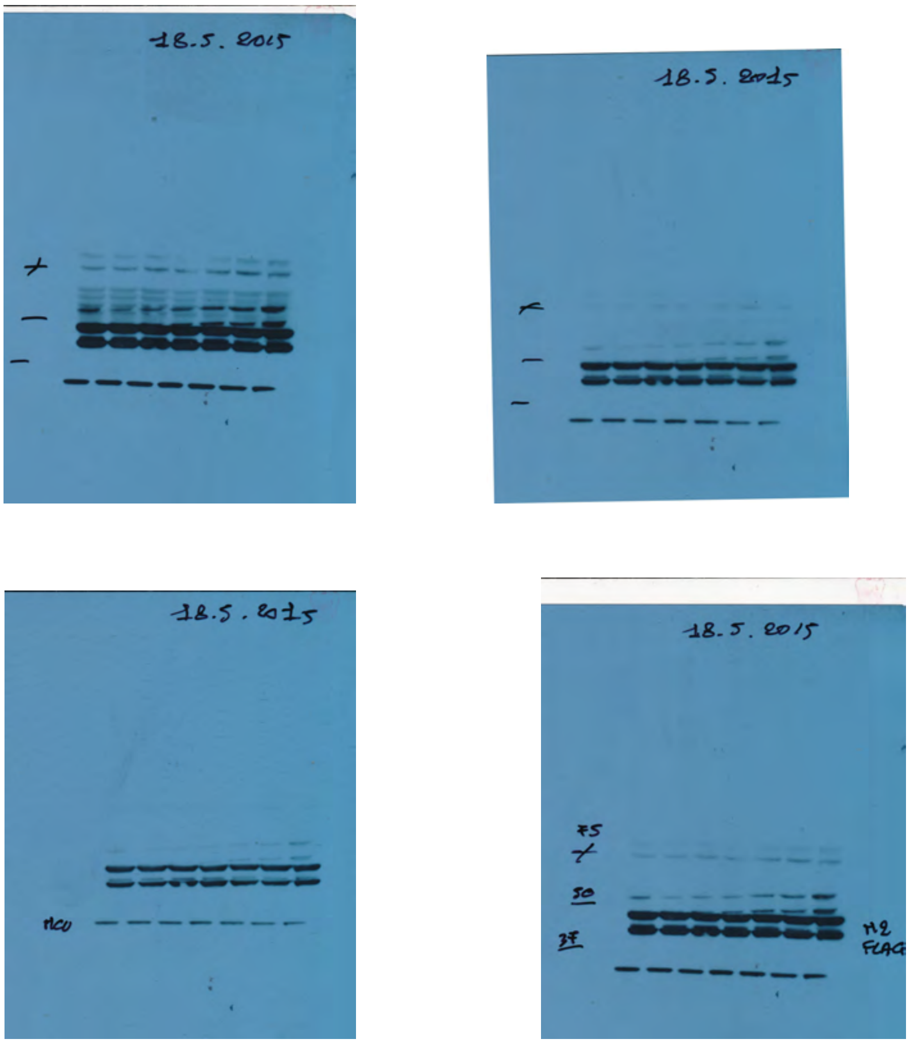

Fig.2A
MICU1

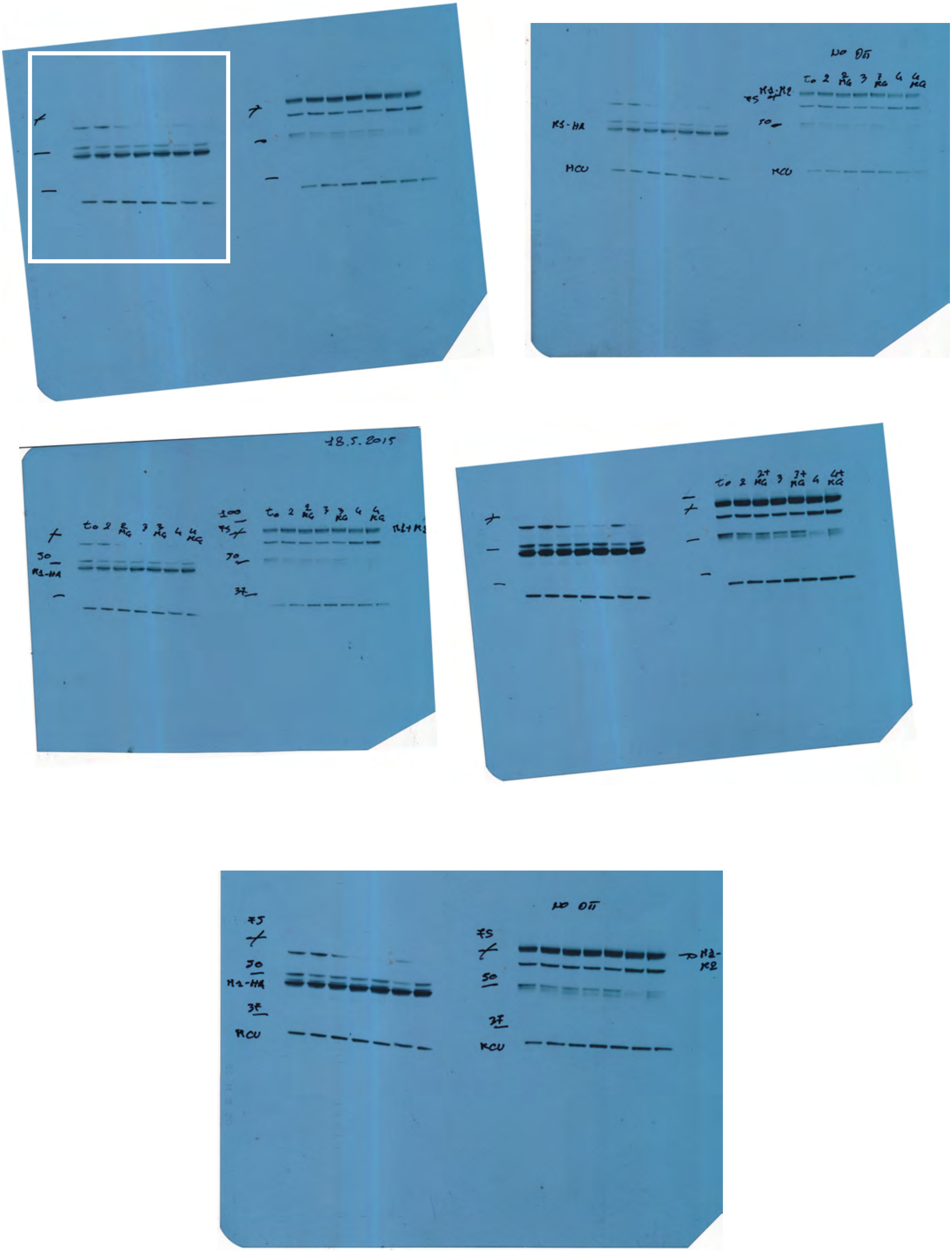

Fig.2A
MICU2

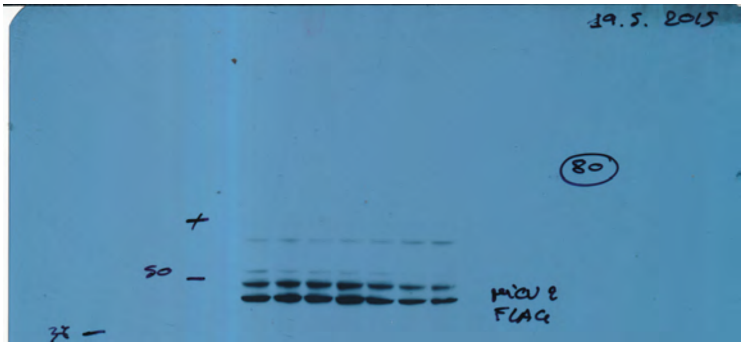

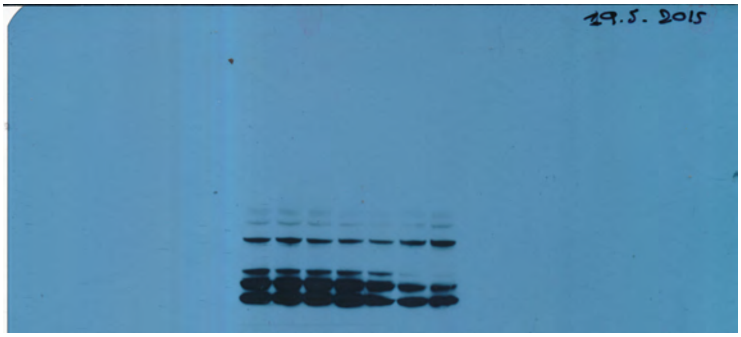

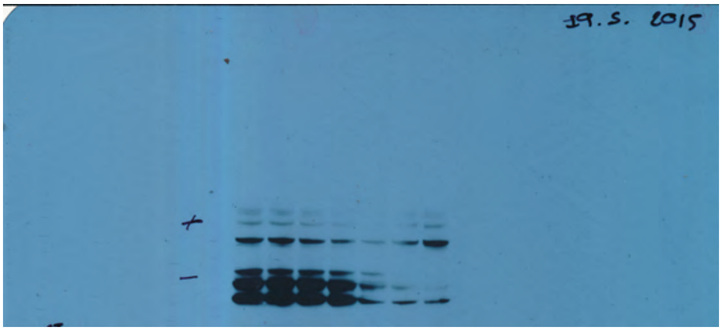

Fig.2D

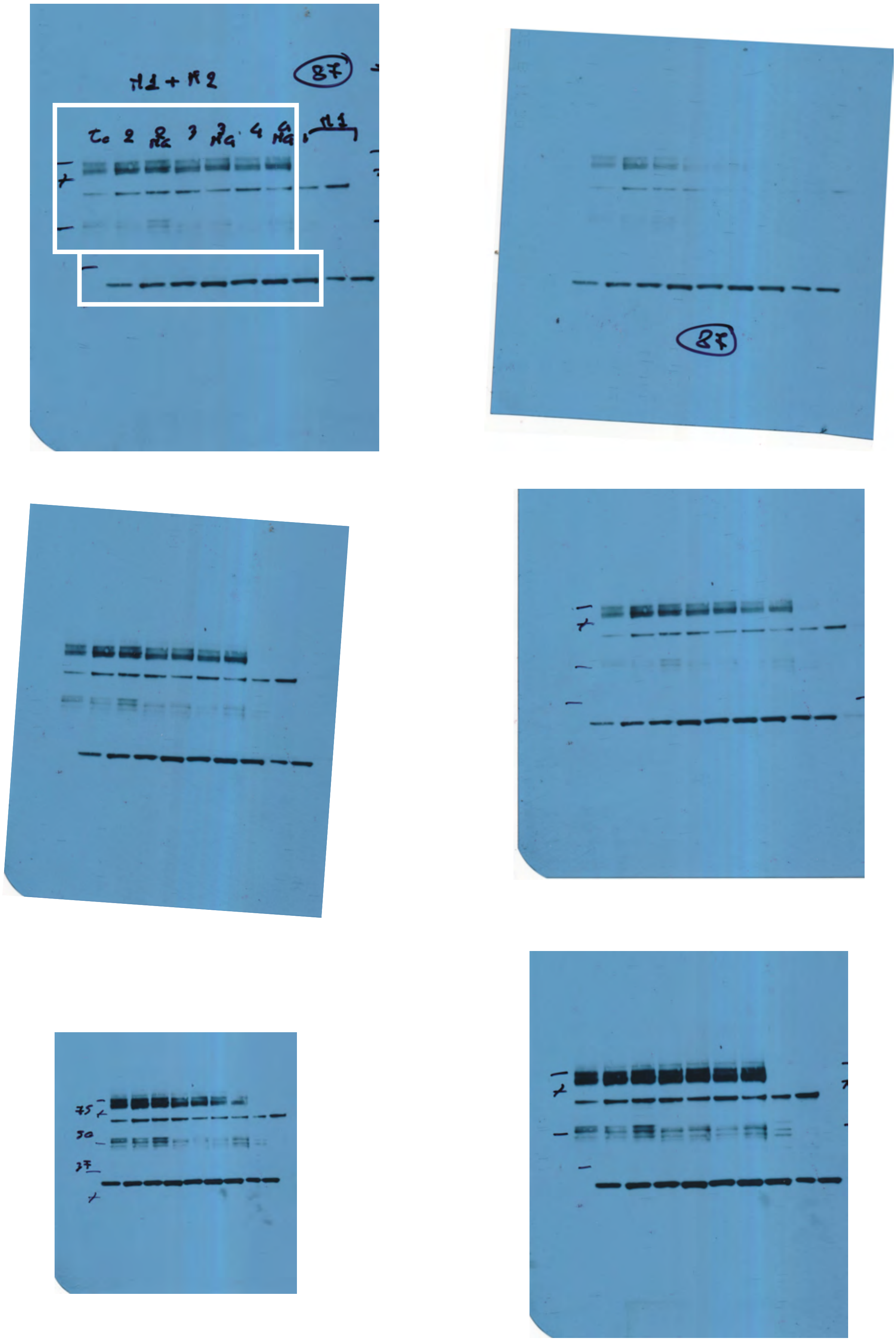

Fig 3
MICU1

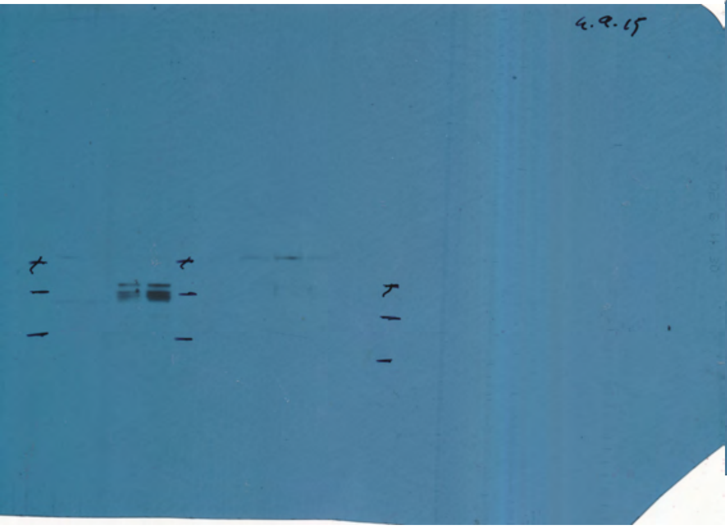
4.9.15

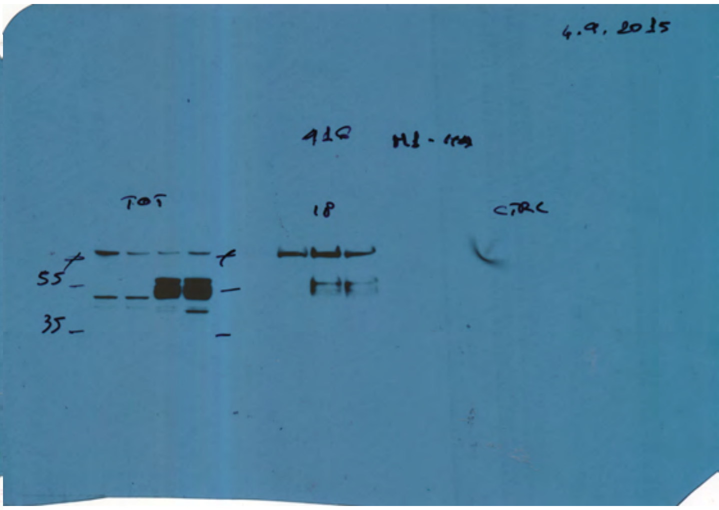
4.9.2015
116
TOT
IP
CTRL
55
35

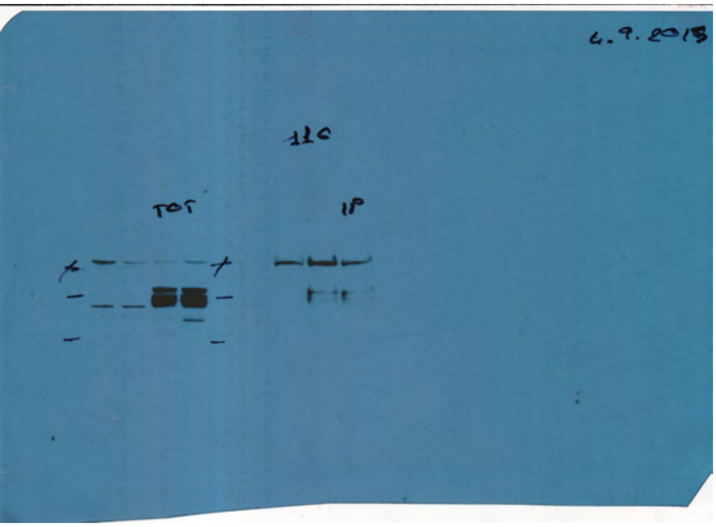
4.9.2015
116
TOT
IP

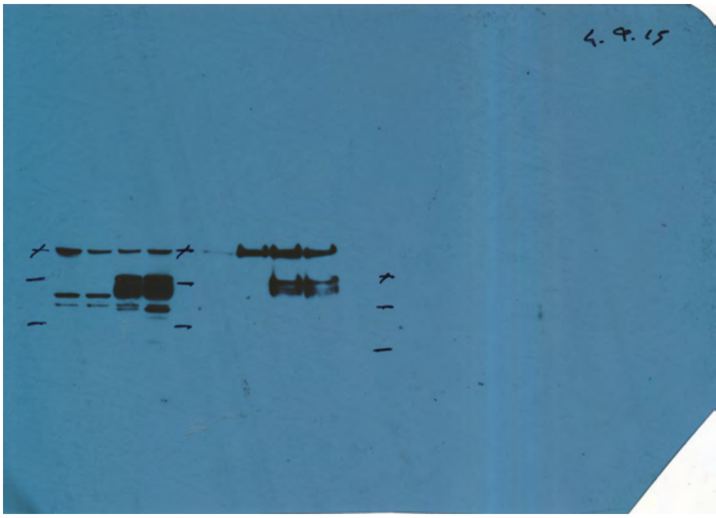
4.9.15

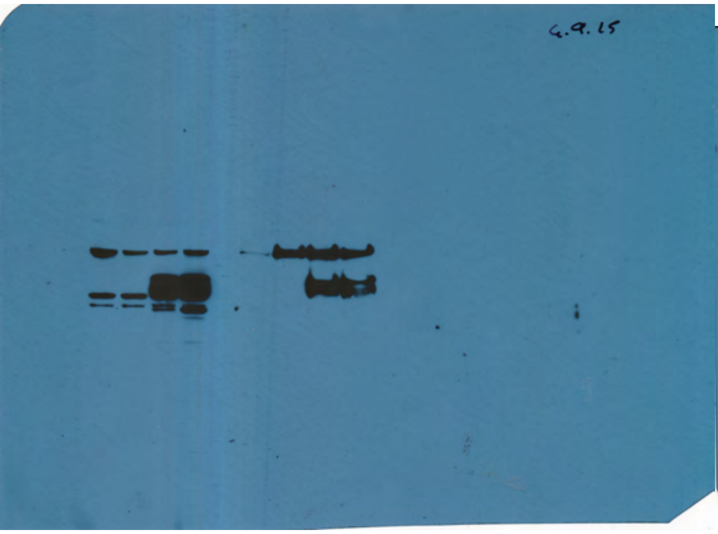
4.9.15

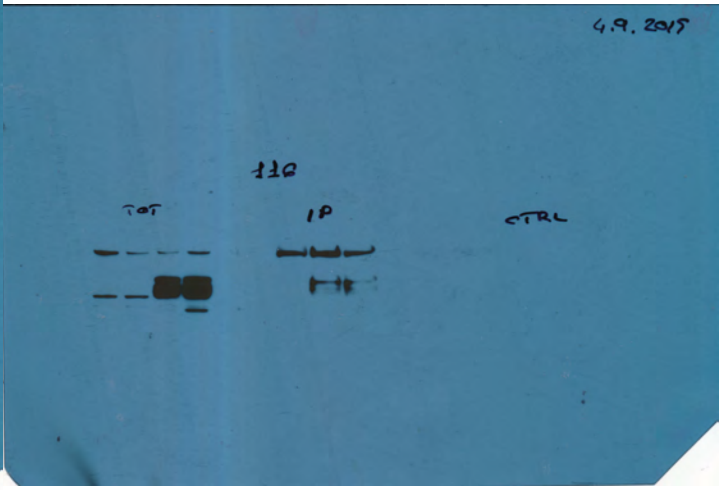
4.9.2015
116
TOT
IP
CTRL

Fig 3
Ub

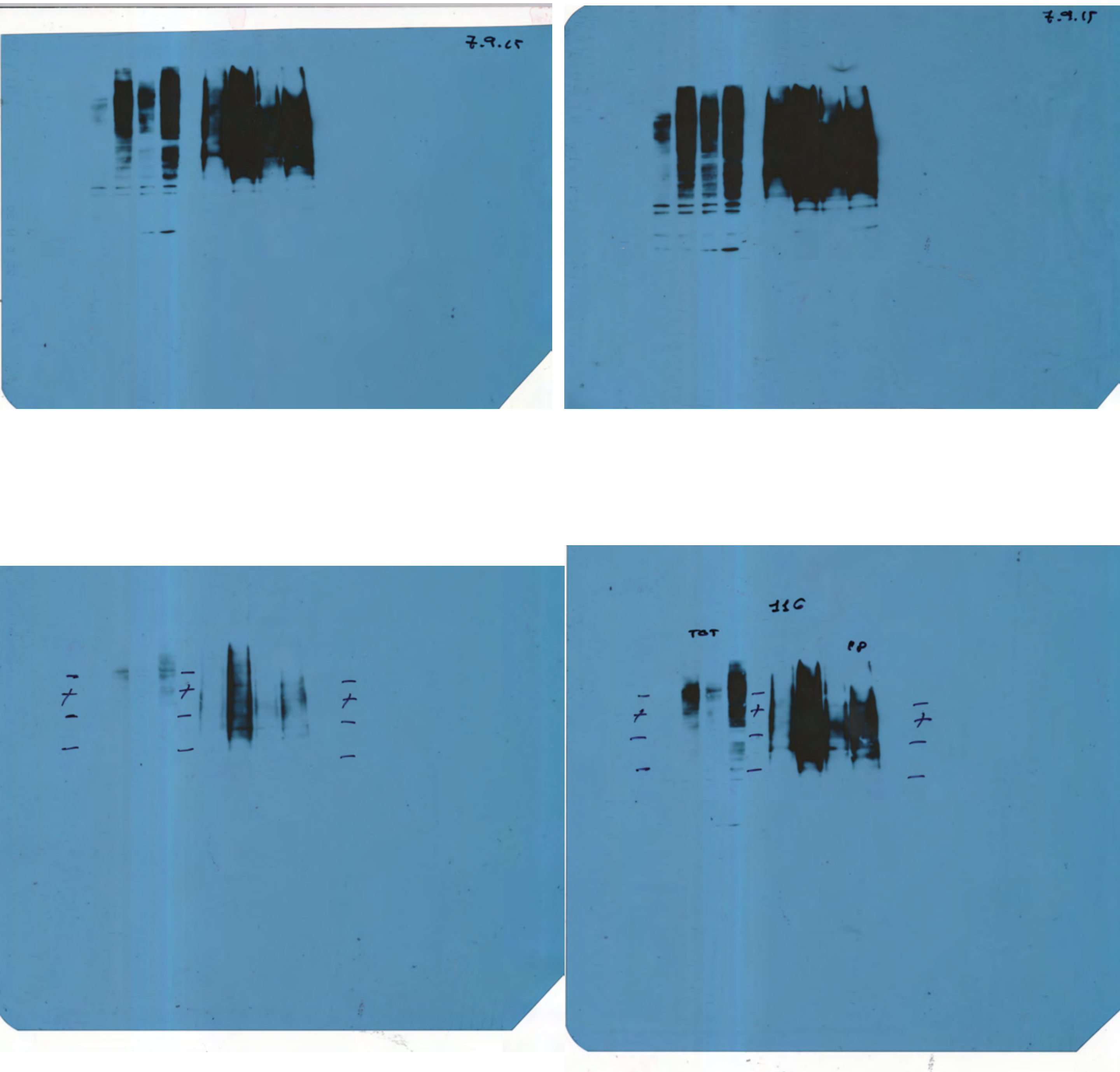

Fig 4A

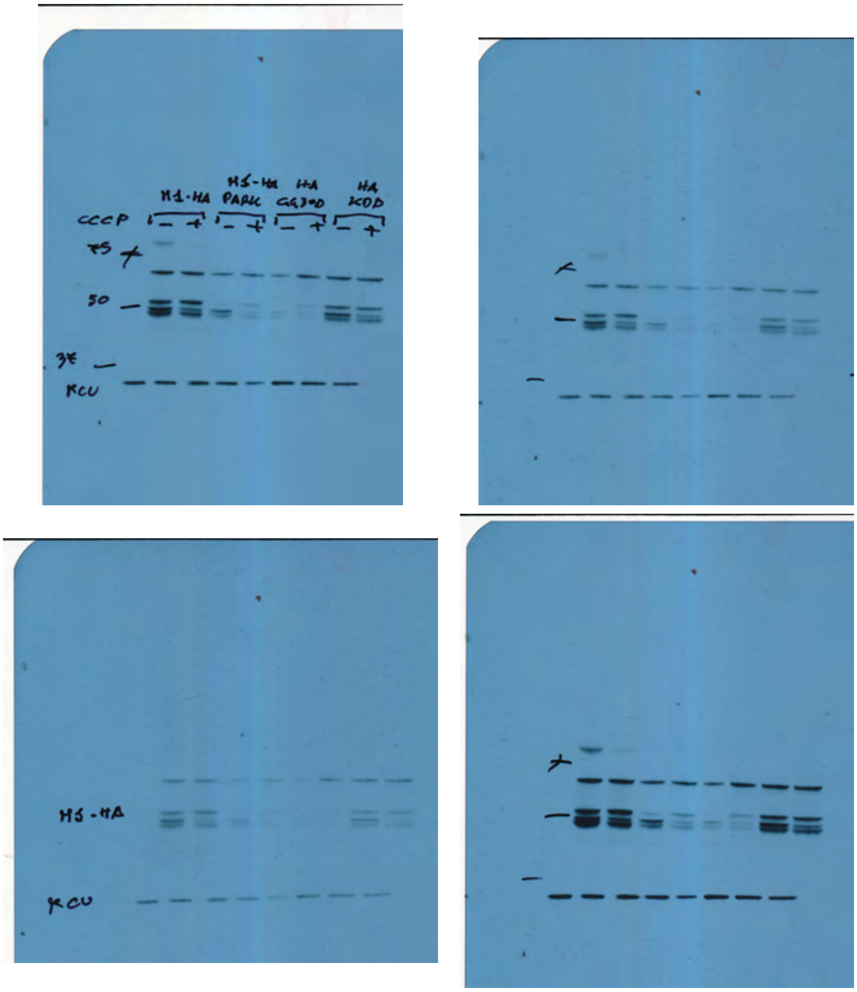

Fig 4C

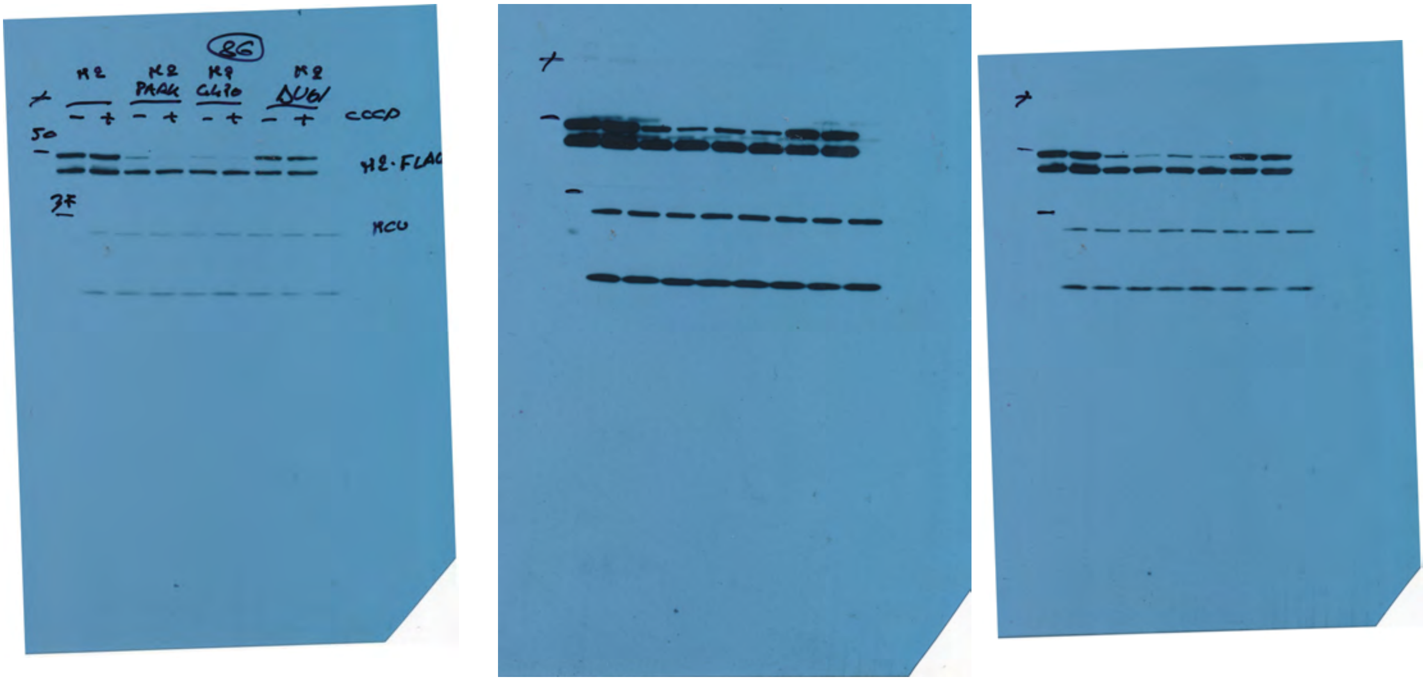

Fig 4E

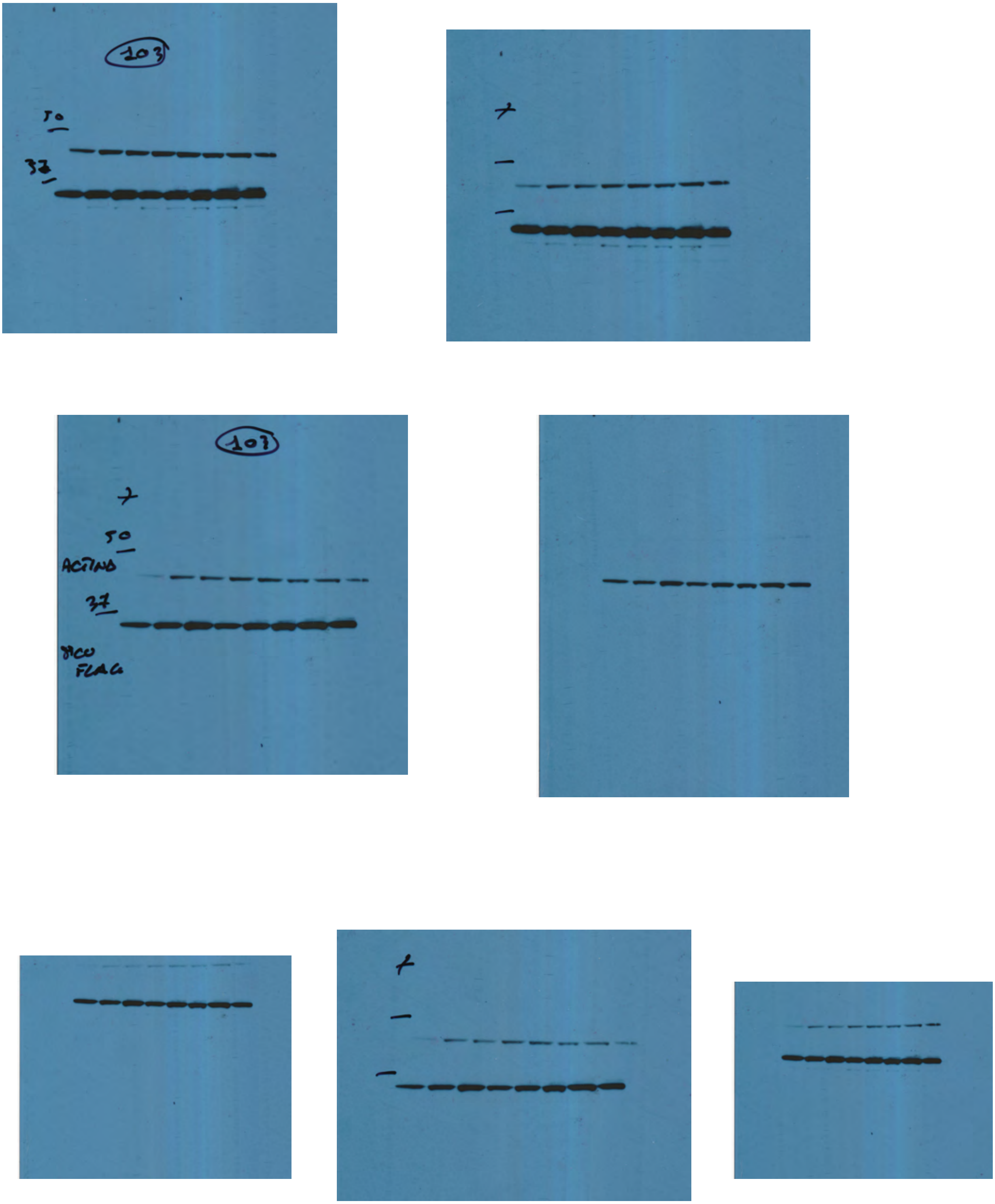

Fig 5A MICU1 e MCU

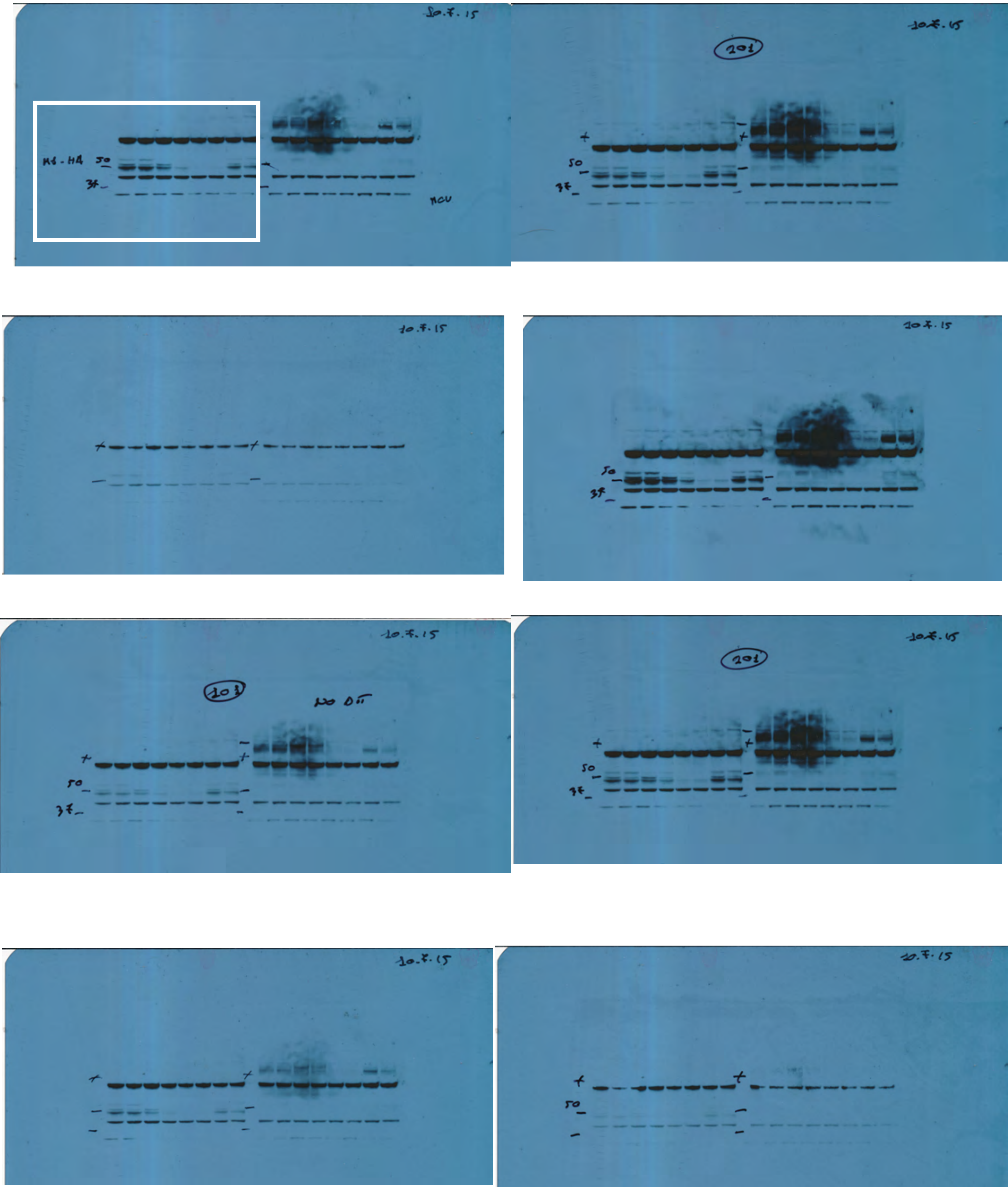

Fig 5A MICU2

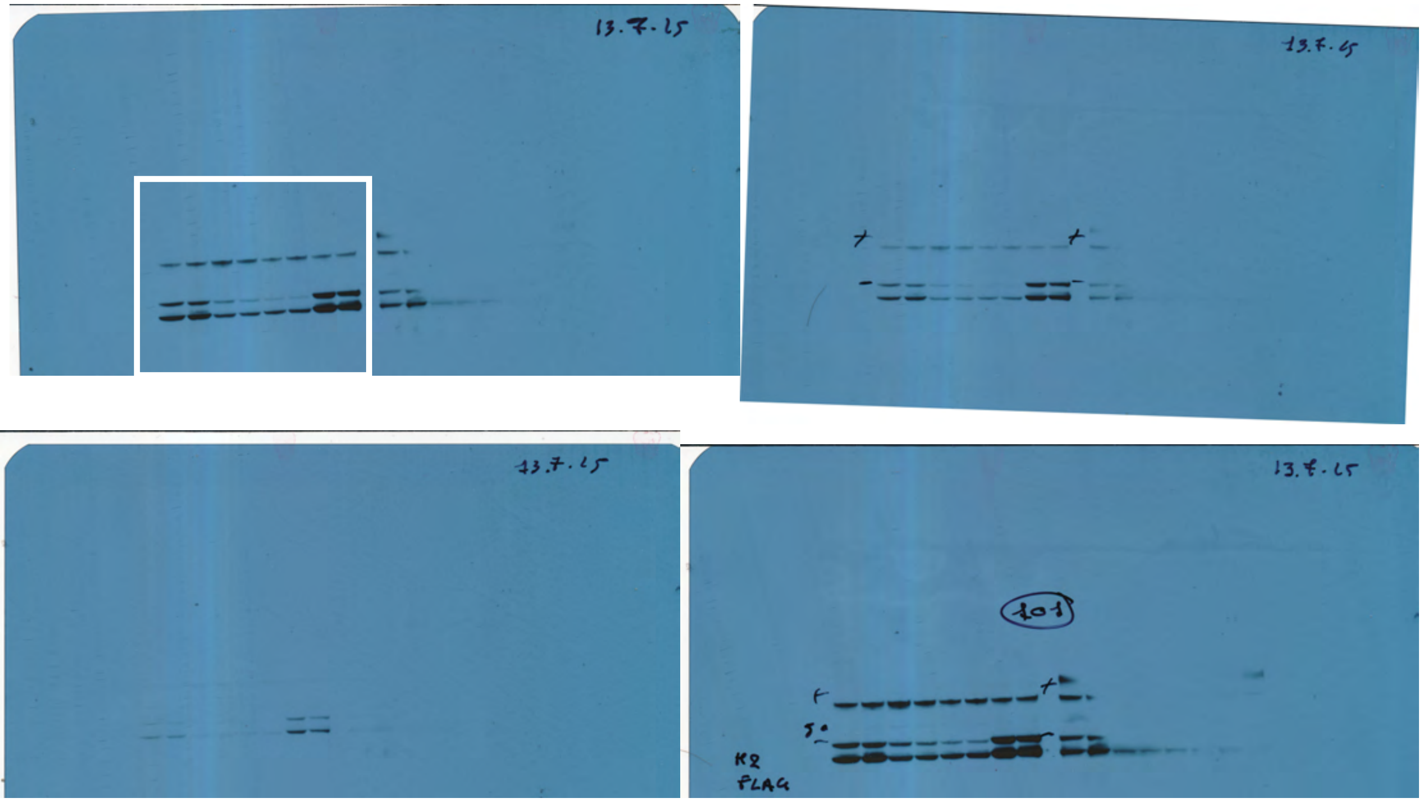

Fig 5D

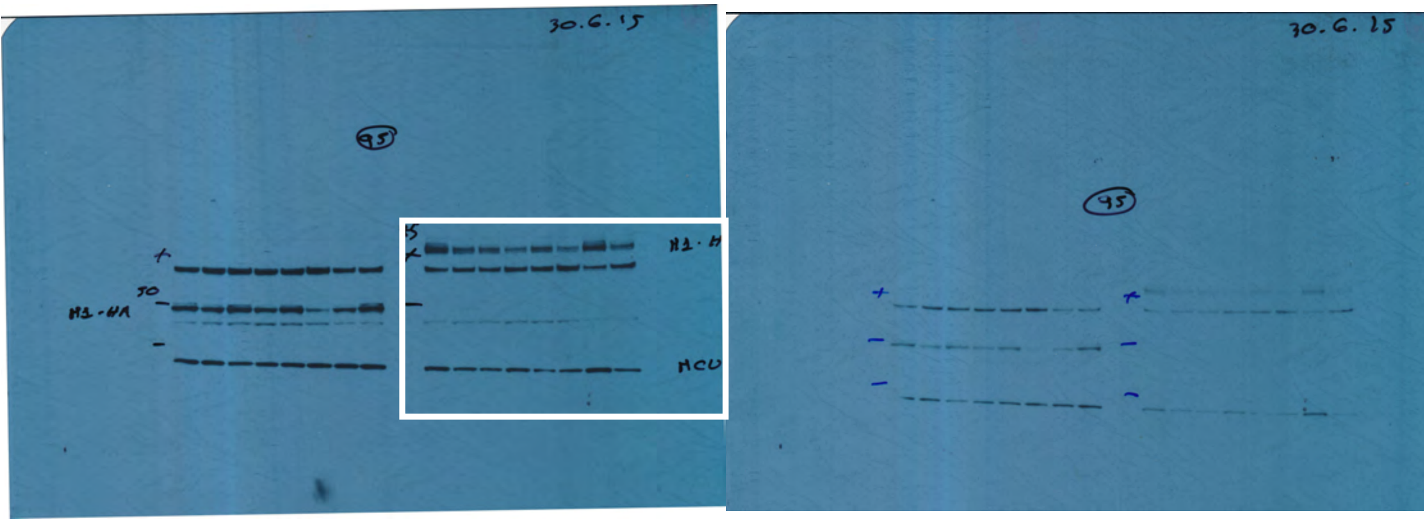

Fig. 6A anti HA

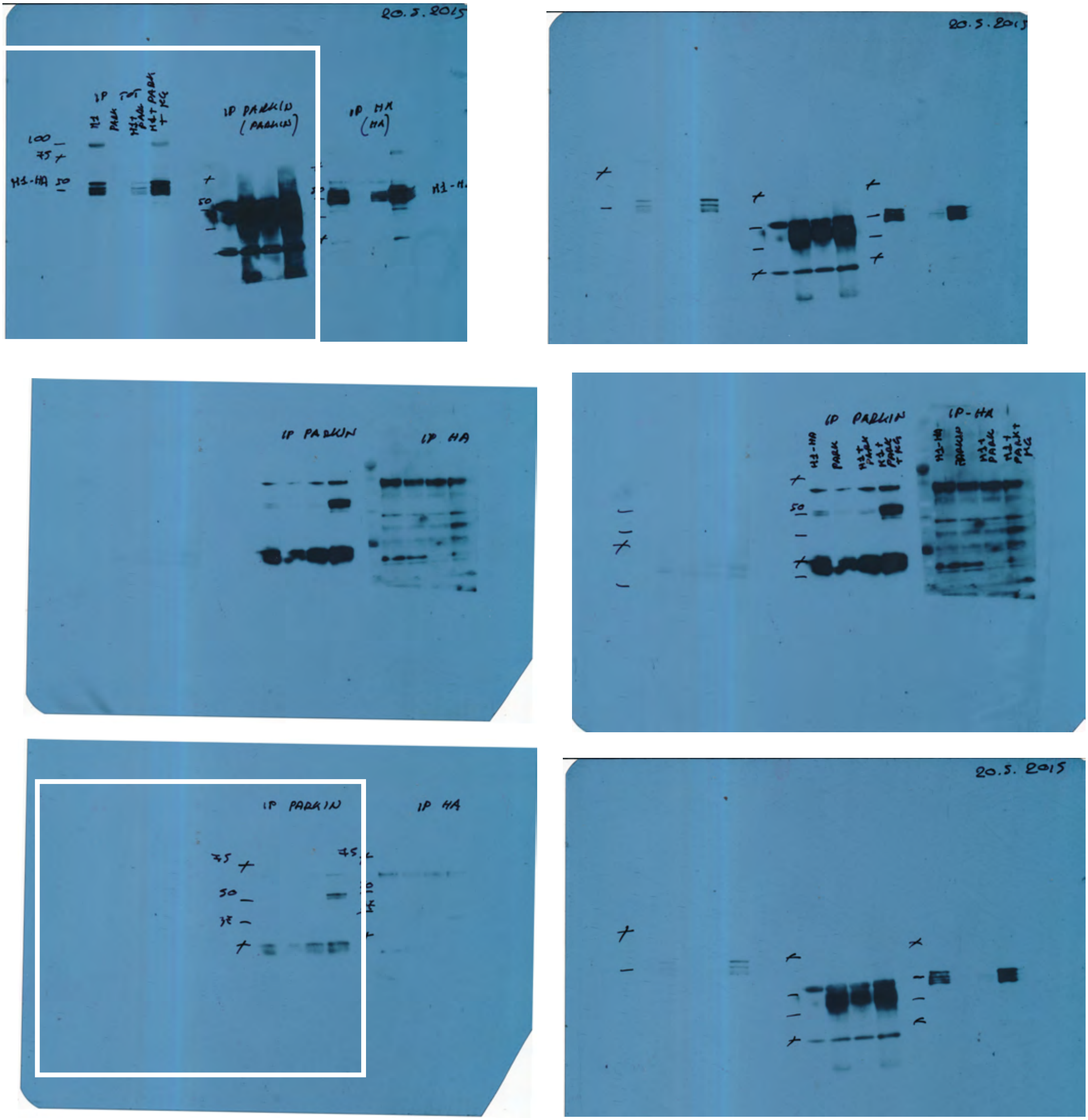

Fig. 6A anti Parkin

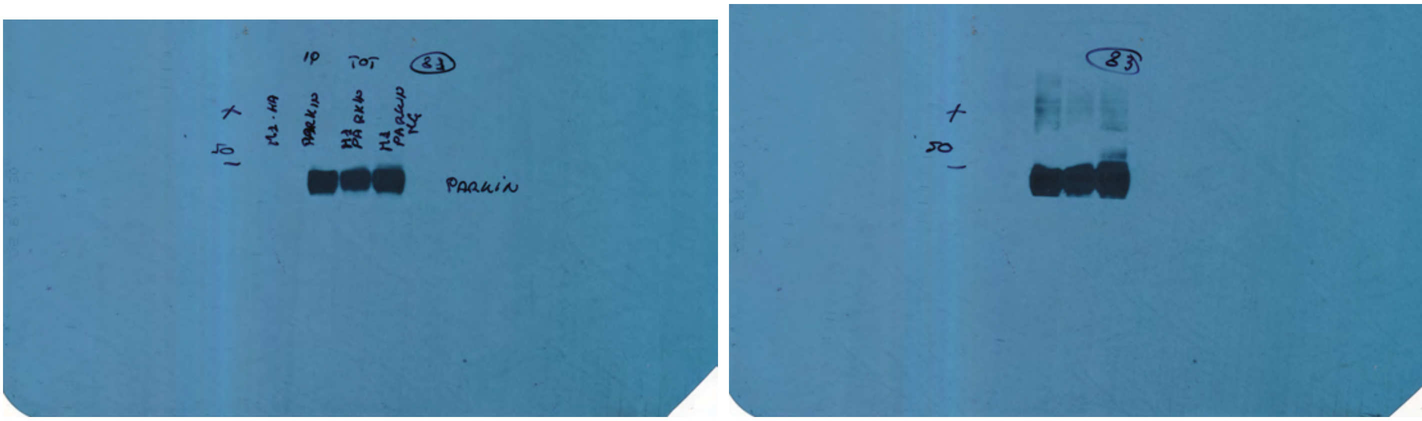

Fig. 6B

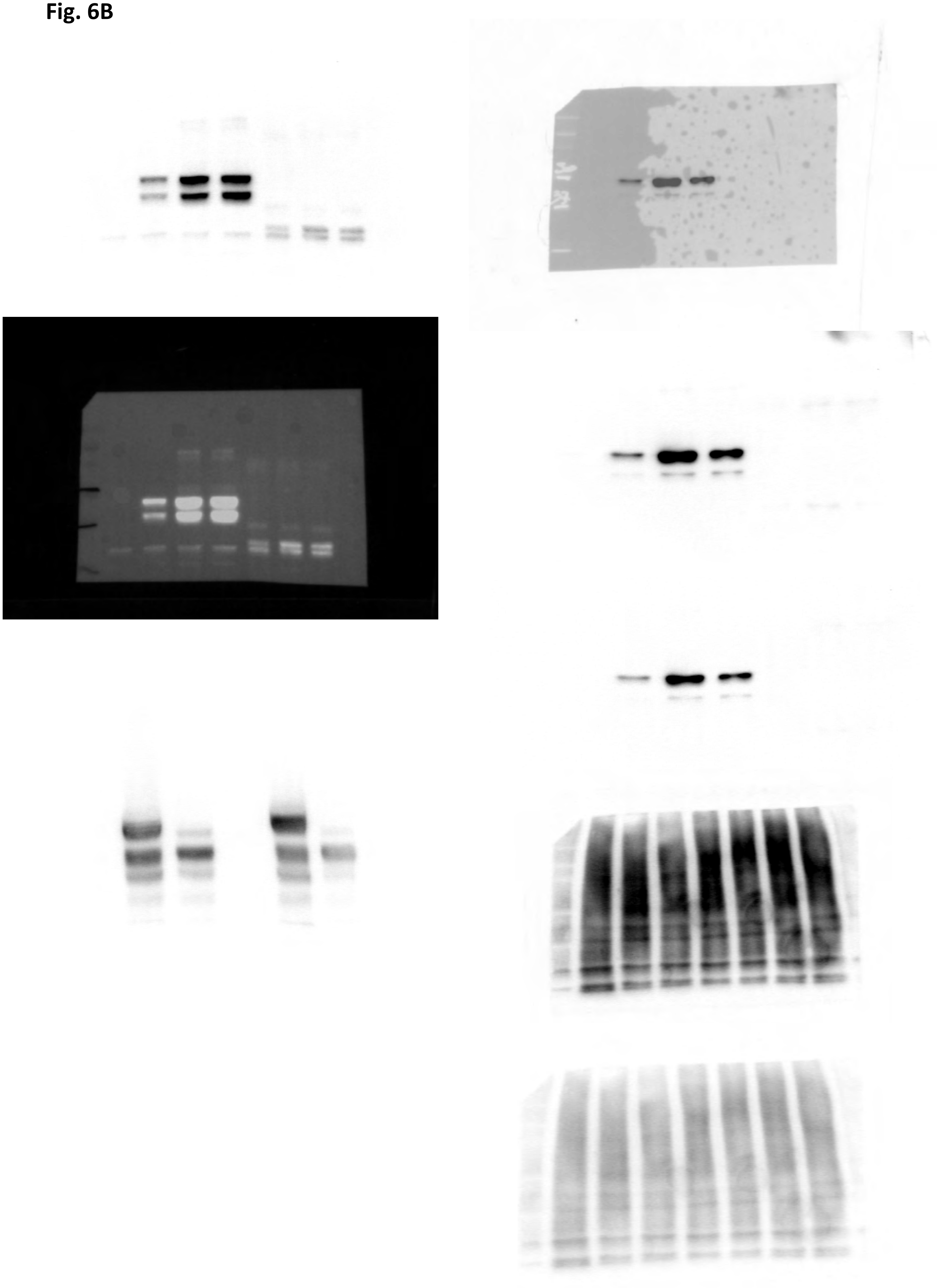

Supplementary 1

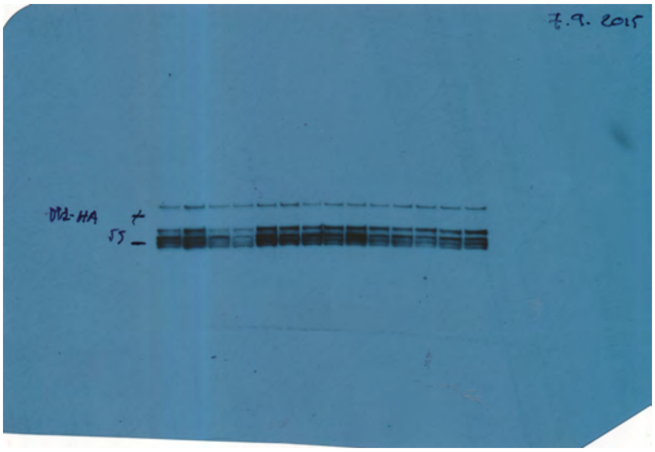

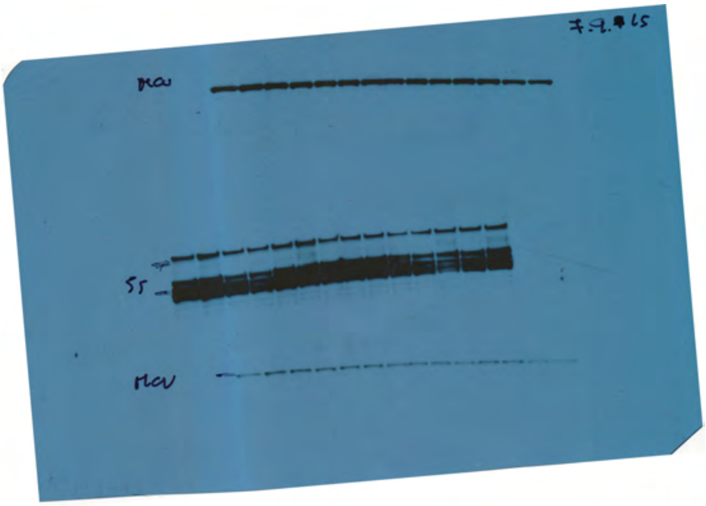

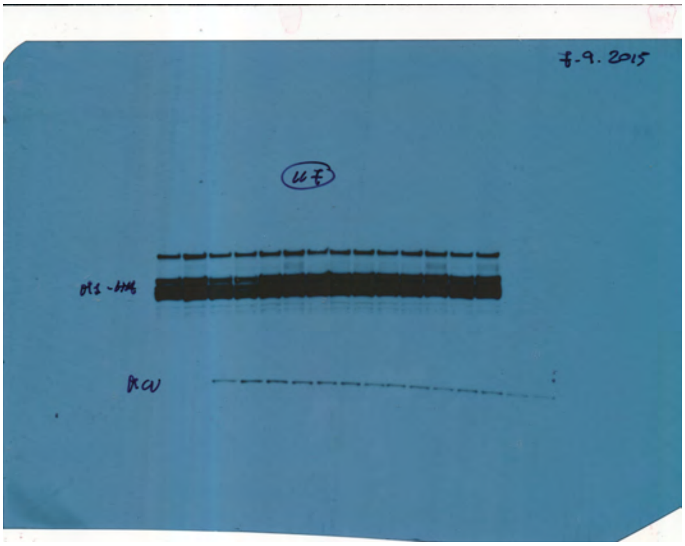

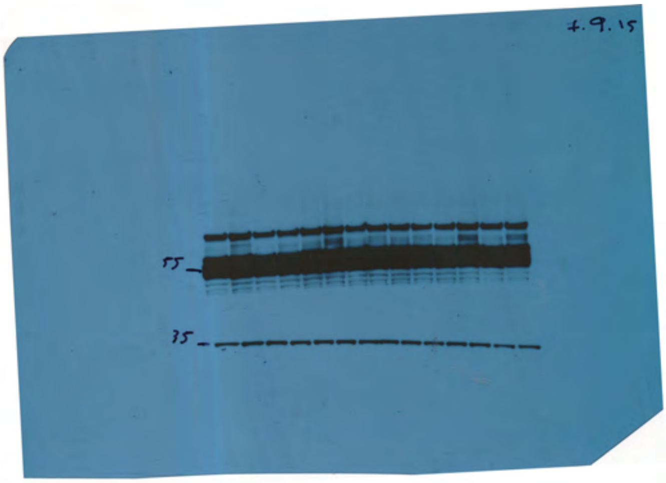

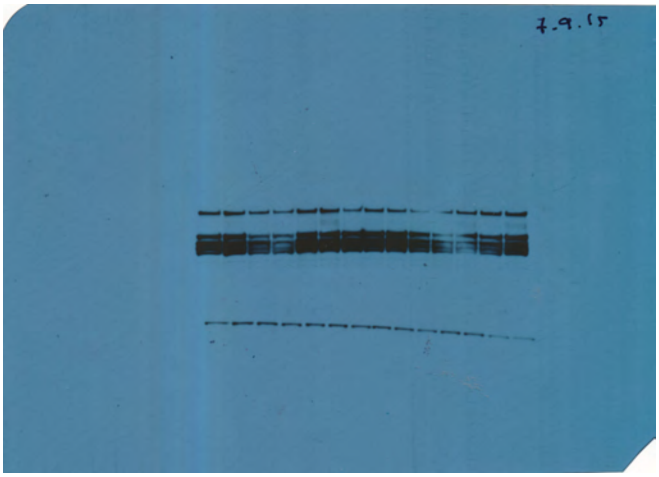

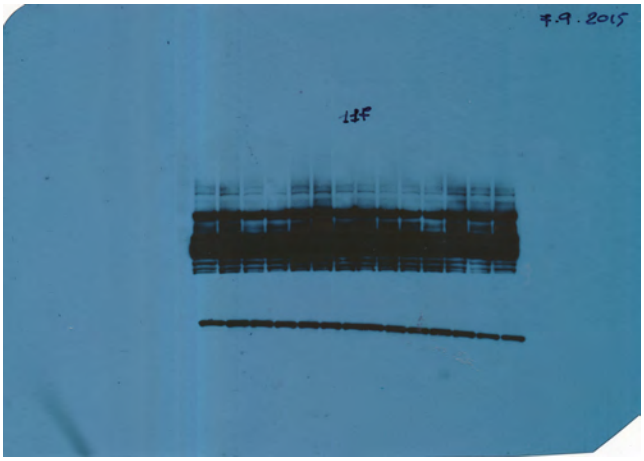

Supplementary 2

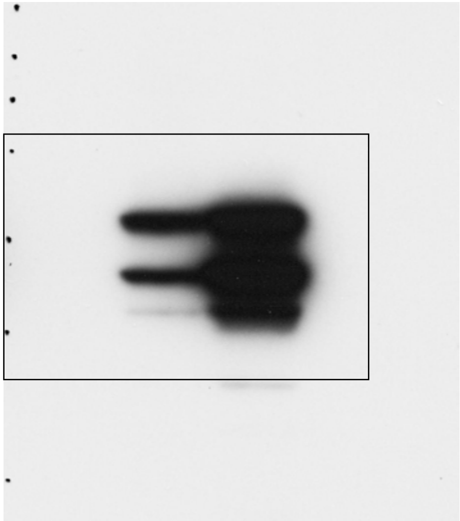

Parkin

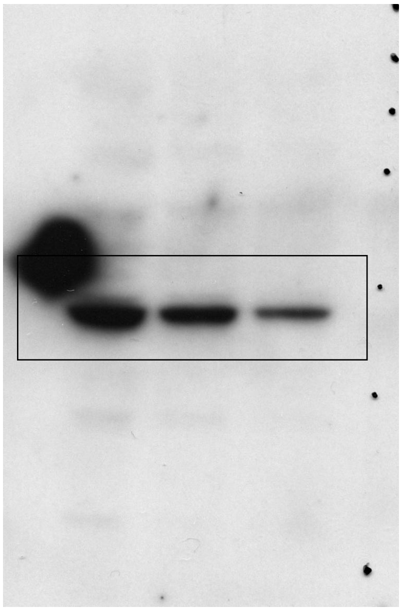

MICU1

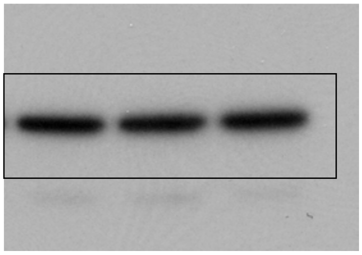

β-actin

MG132

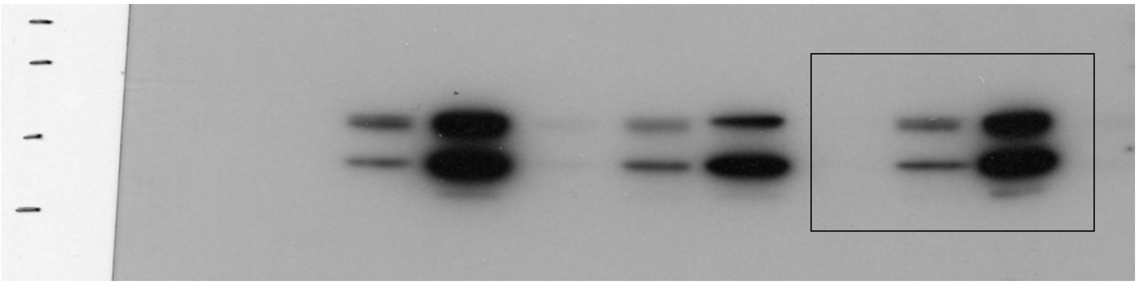

anti-Parkin

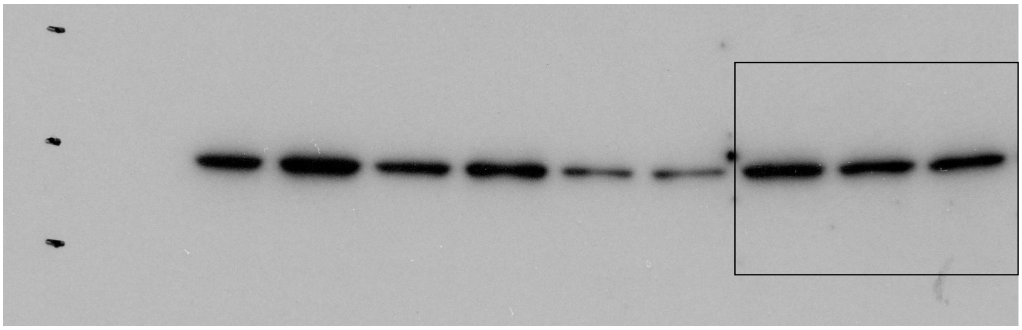

anti-MICU1

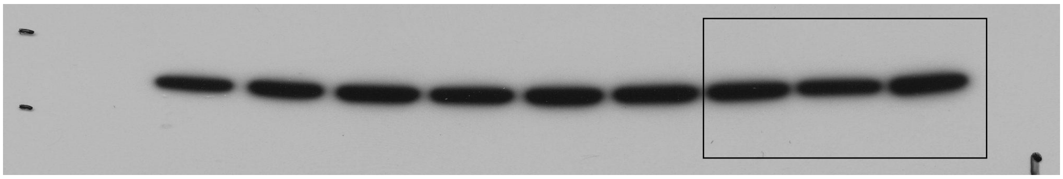

anti-β-actin

Supplementary 3

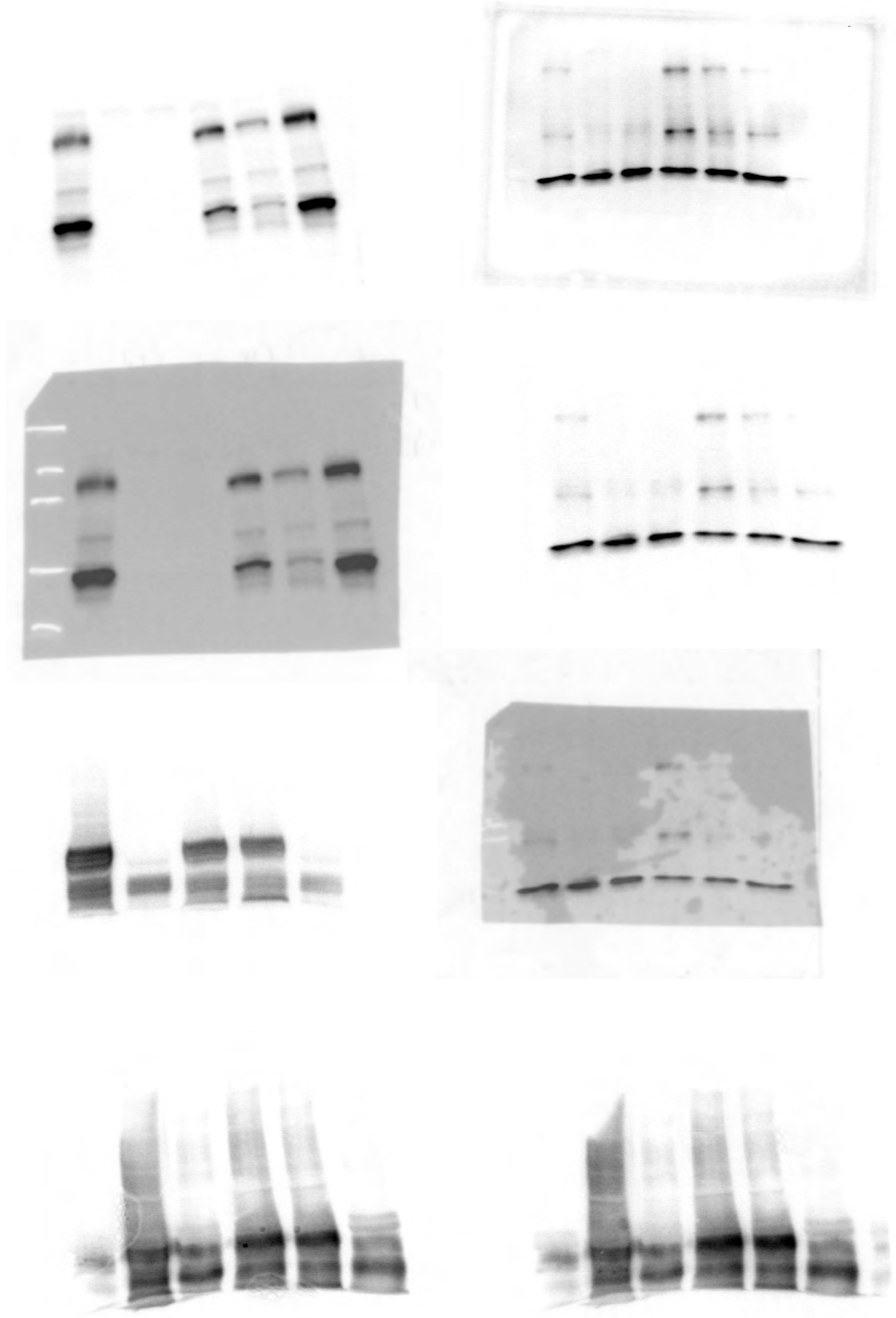

Supplement: Supplementary file 1 — Supplementary information + Full length Blots [file 41598_2018_32551_MOESM1_ESM.pdf]
